# pH-Sensitive Dextrin-Based Nanosponges Crosslinked with Pyromellitic Dianhydride and Citric Acid: Swelling, Rheological Behavior, Mucoadhesion, and In Vitro Drug Release

**DOI:** 10.3390/gels12010090

**Published:** 2026-01-19

**Authors:** Gjylije Hoti, Sara Er-Rahmani, Alessia Gatti, Ibrahim Hussein, Monica Argenziano, Roberta Cavalli, Anastasia Anceschi, Adrián Matencio, Francesco Trotta, Fabrizio Caldera

**Affiliations:** 1 Department of Chemistry, NIS Interdepartmental Centre, University of Turin, Via P. Giuria 7, 10125 Turin, Italy; gjylije.hoti@unito.it (G.H.); sara.errahmani@unito.it (S.E.-R.); ibrahim.mahussein@unito.it (I.H.); anastasiaandrea.anceschi@unito.it (A.A.); fabrizio.caldera@unito.it (F.C.); 2 Department of Drug Science and Technology, University of Turin, Via P. Giuria 9, 10125 Turin, Italy; alessia.gatti@unito.it (A.G.); monica.argenziano@unito.it (M.A.); roberta.cavalli@unito.it (R.C.); 3 Biology Teaching Unit, Department of Biochemistry and Molecular Biology A, Faculty of Veterinary Medicine, Regional Campus of International Excellence “Campus Mare Nostrum”, University of Murcia, 30100 Murcia, Spain; adrian.matencio@um.es

**Keywords:** dextrin, nanosponges, hydrogels, pH-sensitive swelling, Flory–Rehner theory, rheology, mucoadhesion capacity

## Abstract

Dextrin-based nanosponges (D-NS) are promising candidates for oral drug delivery due to their biocompatibility, mucoadhesive properties, and tunable swelling behavior. In this study, pH-sensitive nanosponges were synthesized using β-cyclodextrin (β-CD), GluciDex^®^2 (GLU2), and KLEPTOSE^®^ Linecaps (LC) as building blocks, crosslinked with pyromellitic dianhydride (PMDA) and citric acid (CA). The nanosponges were mechanically size-reduced via homogenization and ball milling, and characterized by FTIR, TGA, dynamic light scattering (DLS), and zeta potential measurements. Swelling kinetics, cross-linking density (determined using Flory–Rehner theory), rheological behavior, and mucoadhesion were evaluated under simulated gastric and intestinal conditions. The β-CD:PMDA 1:4 NS was selected for drug studies due to its optimal balance of structural stability, swelling capacity (~863% at pH 6.8), and highest apomorphine (APO) loading (8.23%) with 90.58% encapsulation efficiency. All nanosuspensions showed favorable polydispersity index values (0.11–0.30), homogeneous size distribution, and stable zeta potentials, confirming suspension stability. Storage at 4 °C for six months revealed no changes in physicochemical properties or apomorphine (APO) degradation, indicating protection by the nanosponge matrix. D-NS exhibited tunable swelling, pH-responsive behavior, and mucoadhesive properties, with nanoparticle–mucin interactions quantified by the rheological synergism parameter (∆G′ = 53.45, ∆G″ = −36.26 at pH 6.8). In vitro release studies demonstrated slow, sustained release of APO from D-NS in simulated intestinal fluid compared to free drug diffusion, highlighting the potential of D-NS as pH-responsive, mucoadhesive carriers with controlled drug release and defined nanoparticle–mucin interactions.

## 1. Introduction

Neurodegenerative diseases, including Alzheimer’s disease (AD), Parkinson’s disease (PD), multiple sclerosis, amyotrophic lateral sclerosis, and Huntington’s disease, are progressive disorders characterized by the gradual loss of neuronal structure and function. Their global prevalence is rising, largely due to increased life expectancy and the accumulation of biological stressors such as oxidative damage, chronic inflammation, mitochondrial dysfunction, protein aggregation, and environmental factors. Despite advances in therapeutic research, effective long-term management remains challenging, particularly because many neuroprotective and symptomatic agents exhibit poor pharmacokinetic profiles and limited ability to reach the central nervous system [1,2,3,4].

Nanosized drug delivery systems have emerged as promising tools for improving therapeutic efficacy in neurodegenerative disorders [5]. Their small dimensions, tunable surface chemistry, and ability to modulate drug release make them advantageous for crossing biological barriers, including the blood–brain barrier (BBB), and for protecting labile drugs from degradation [6,7]. Among noninvasive strategies, oral delivery remains the most convenient and patient-friendly route, especially for chronic conditions [8,9]. Orally administered drugs must inevitably pass through the stomach before reaching the small intestine or colon, the primary sites of absorption. The gastrointestinal (GI) tract presents formidable physiological, enzymatic, and mechanical barriers, including wide pH variations, digestive enzymes, mucus turnover, peristalsis, and limited epithelial permeability, all of which can compromise drug absorption and stability. Consequently, many drugs suffer from degradation, poor solubility, or limited permeability, resulting in low oral bioavailability [10,11,12,13,14,15,16].

Mucoadhesive drug delivery systems offer an attractive approach to overcome these limitations. By adhering to the mucus layer that lines the GI tract, mucoadhesive polymers prolong the residence time of the formulation at the absorption site, enhance drug stability and bioavailability, and enable sustained release [17,18]. These systems can be engineered to target specific GI regions and can also facilitate transmucosal absorption, thereby bypassing hepatic first-pass metabolism [19,20]. A suitable polymeric material for mucoadhesive formulations should exhibit the following characteristics: (i) the presence of strong anionic or cationic functional groups; (ii) sufficiently high molecular weight; (iii) favorable interfacial properties for mucus penetration; (iv) high polymer chain mobility; (v) high drug-loading capability; (vi) pronounced swelling behavior in aqueous media; (vii) strong interaction with the mucosal layer; (viii) allow prolonged release of therapeutic agents; and (ix) biodegradability [21].

Hydrophilic polymers containing hydroxyl, carboxyl, or amino groups, such as chitosan, pectin, alginate, and cellulose derivatives, are commonly employed due to their capacity to interact with mucin glycoproteins through hydrogen bonding and electrostatic interactions [22]. Nevertheless, many conventional mucoadhesive polymers suffer from limitations related to pH sensitivity, poor mechanical stability, or reduced biodegradability, which may restrict their long-term applicability. As detailed by Twana Mohammed M. Way et al., although chitosan has been extensively explored as a mucoadhesive polymer, its limited solubility and reduced mucoadhesive performance at neutral and basic pH significantly constrain its effectiveness under physiological conditions. Numerous chitosan derivatives such as quaternized, thiolated, carboxymethylated, and cyclodextrin-grafted chitosans have been developed to address these limitations; however, their synthesis often involves complex chemical modifications that raise concerns regarding reproducibility, stability, and scalability, highlighting the need for alternative, more robust mucoadhesive platforms [23]. Also, chitosan, carries a high density of positive charges and may disrupt cell membranes by interacting with the negatively charged bilayer [24]. Myung-Kwan Chun et al. reported that poly(vinyl pyrrolidone) (PVP)–poly(acrylic acid) (PAA) interpolymer complexes exhibit enhanced mucoadhesion and reduced solubility under acidic conditions due to hydrogen bonding, making them suitable for gastric transmucosal drug delivery. However, their performance remains strongly pH-dependent, with limited applicability outside acidic environments, and relies on non-covalent interactions that may be destabilized under physiological conditions. In addition, rapid dissolution or erosion at higher pH and restricted control over long-term drug release limit their broader transmucosal applicability [25]. As mentioned, first-generation mucoadhesive polymers, including cationic chitosan and anionic PAA, rely on non-covalent interactions, making them highly sensitive to pH, ionic strength, and mucus turnover, which limits residence time and drug delivery efficiency. Second-generation systems, such as lectins or thiolated polymers, provide stronger, targeted adhesion via covalent or receptor-mediated interactions; however, many lectins are immunogenic or toxic, and thiomers may offer limited control over drug release due to increased crosslinking and rigidity [26]. To address these challenges, increasing attention has been directed toward polysaccharide-based carriers, particularly dextrin and cyclodextrin derivatives. These starch-based materials are biodegradable, biocompatible, and structurally versatile, enabling precise modulation of physicochemical properties through chemical modification or crosslinking [27].

Cyclodextrins (CDs) are cyclic oligosaccharides composed of α-(1,4)-linked D-glucopyranose units, characterized by a hydrophilic outer surface and a lipophilic internal cavity that enables host–guest inclusion complex formation. Linecaps (LC) and Glucidex^®^ (GLU2) are linear dextrins (maltodextrins) derived from starch hydrolysis, consisting mainly of α-(1,4)-linked glucose units and exhibiting strong hydration, swelling, and intrinsic mucoadhesive behavior. While dextrins provide structural hydration and adhesion, CDs enhance drug solubility and protect labile compounds from oxidative or enzymatic degradation through inclusion complexation. Polymerization of cyclodextrin and dextrin units using multifunctional crosslinking agents yields cyclodextrin-based nanosponges, three-dimensional, crosslinked networks with tunable swelling, pH-responsive behavior, and high drug-loading capacity. These combined properties make dextrin- and cyclodextrin-based nanosponges particularly suitable for oral drug delivery systems requiring controlled release, enhanced stability, and protection against harsh gastrointestinal conditions [28,29,30,31,32,33,34,35,36]. The pH-responsive behavior of these polymers arises from the presence of ionizable functional groups, such as carboxyl (–COOH) and hydroxyl (–OH) moieties. pH-responsive polymers are considered smart biomaterials, as they can be rationally designed to exhibit site-specific responses to defined pH ranges, enabling controlled drug release through pH-dependent swelling [37]. pH-responsive polymers contain ionizable functional groups, such as –COOH and –OH, which enable site-specific responses to changes in pH through swelling, thereby allowing controlled drug release. Although pH-sensitive hydrogels are widely used in drug delivery and other applications, challenges remain in achieving stable performance across acidic and basic conditions, maintaining mechanical stability during swelling, and ensuring degradation within a desired timeframe [38,39]. Apomorphine (APO), a potent dopamine agonist used in the management of Parkinson’s disease, exemplifies the challenges associated with oral delivery. The molecule is chemically unstable, undergoing rapid oxidative degradation in aqueous media, and shows extreme sensitivity to pH, temperature, and light. Moreover, APO exhibits very low oral bioavailability (<4%) due to extensive hepatic first-pass metabolism, necessitating frequent subcutaneous injections or continuous infusion. These administration strategies are burdensome for patients and may negatively affect compliance, especially in advanced stages of the disease. A mucoadhesive, pH-responsive delivery platform capable of stabilizing APO and sustaining its release through the GI tract could significantly improve therapeutic management [40,41,42].

In this context, as physiological conditions are associated with site-specific pH variations, dextrin-based nanosponges crosslinked using pyromellitic dianhydride (PMDA) and citric acid (CA) offer a promising alternative. Their hydrophilic, multifunctional network, rich in hydroxyl and carboxyl groups, supports strong swelling, water uptake, and mucoadhesion. By modulating the dextrin-to-crosslinker molar ratios, it is possible to finely tune network architecture, cross-linking density, rheological behavior, and pH sensitivity. These properties are essential for designing oral delivery carriers that can withstand gastric conditions while enabling targeted release in the intestinal environment.

This study aims to synthesize and characterize pH-sensitive dextrin-based nanosponges crosslinked with PMDA and CA and to evaluate their swelling behavior, rheological properties, mucoadhesive performance, and in vitro drug release. The goal is to establish a polymeric platform suitable for oral administration of unstable or poorly bioavailable drugs, with particular relevance to therapeutic agents used in neurodegenerative diseases such as PD.

## 2. Results and Discussion

### 2.1. Confirmation of Cross-Linking (FTIR and TGA)

Dextrin-based nanosponges (D-NS) are synthesized successfully by cross-linking the hydroxyl groups of β-cyclodextrin (β-CD) with pyromellitic dianhydride (PMDA) and citric acid (CA), as illustrated in Figure S1. For the β-CD:PMDA nanosponge, the synthesis proceeded through a ring-opening reaction of PMDA. The anhydride ring was opened by a nucleophilic attack from the hydroxyl groups on the β-CD structure, facilitated by triethylamine (Et_3_N) as a catalyst [33]. This reaction led to the formation of carboxyl and ester groups within the polymer network. Citric acid (CA), a non-toxic cross-linking agent widely used for cross-linking starch, was also selected for cross-linking with β-CD. The β-CD:CA nanosponge was synthesized through the formation of a five-membered cyclic anhydride intermediate [43]. During the initial step of cross-linking β-CD with CA, CA undergoes dehydration upon heating, resulting in water loss and formation of the cyclic anhydride. Esterification of β-CD with CA, catalyzed by sodium hypophosphite monohydrate (SHP), occurs efficiently at temperatures below 140 °C. This reaction occurred as what has already been noticed before. The formation of synthesized D-NS at different ratios between the building block and cross-linking agent is confirmed by Fourier-transform infrared spectra (FTIR). The broad peak appearing at 3412 cm^−1^ (Figures S2 and S3) shows the presence of OH groups in unmodified Dextrins (ν (CH-OH) and (CH_2_-OH)). The band at 1638 cm^−1^ corresponds to the deformation of primary and secondary OH groups. Further in Figure S2, the absorption band of the carbonyl group (C=O stretching) of PMDA appears at 1771 cm^−1^. The polymerization reaction is confirmed by the shifting of the carbonyl group (C=O stretching) to 1723 cm^−1^. The occurrence of a C=O peak at 1720 cm^−1^, in the FTIR spectrum, is the characteristic feature of PMDA-based D-NS (Figure S2) and CA-based D-NS (Figure S3). In the spectrum of CA as the cross-linking agent (Figure S3), the absorptions at 1728 cm^−1^ and 1371 cm^−1^ are assigned to the C=O stretching vibration, and at 1624 cm^−1^ due to the carboxylic acid moieties.

Thermogravimetric analysis (TGA) is used to assess the thermal stability of synthesized D-NS. The second weight losses, occurring above 240 °C (Figures S4 and S5), are related to the maximum degradation process of the cross-linked structure of D-NS. This indicates good thermal stability of D-NS (PMDA-based D-NS, and CA-based D-NS). The formation of D-NS can be explained by their lower thermal stability compared to the native dextrins (βCD, LC, and GLU_2_) due to the presence of weaker bonds in modified dextrins (D-NS). The changes in the thermograms of the D-NS from the native dextrins are due to changes in their chemical structures.

### 2.2. Swelling Behavior at Different pH

**Figure 1:**
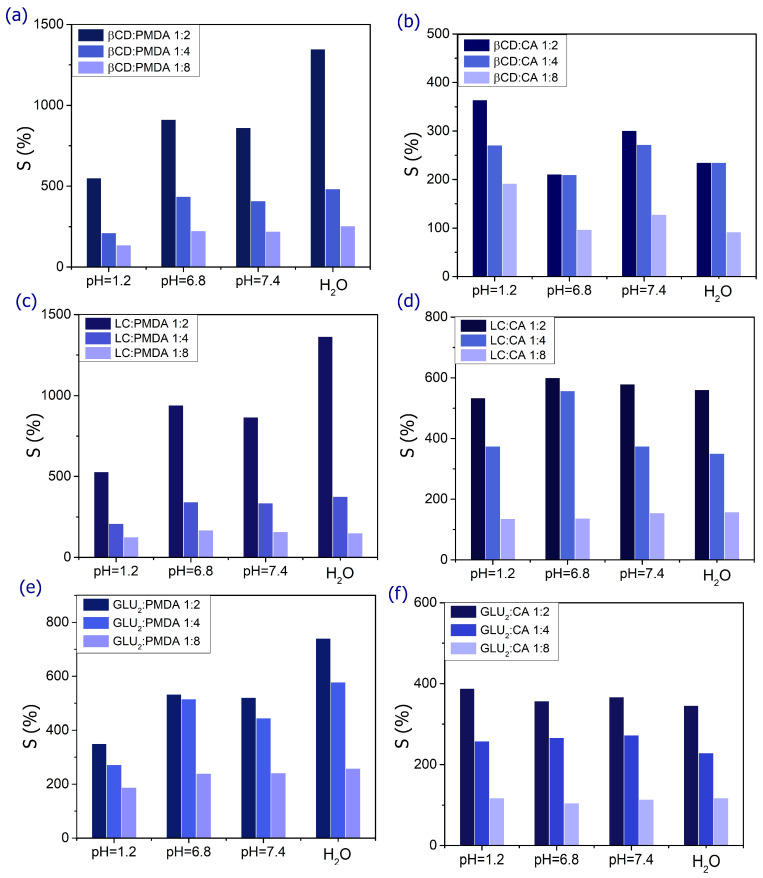
Swelling (S, %) of D-NS: (**a**) β-CD:PMDA (1:2, 1:4, 1:8), (**b**) β-CD:CA (1:2, 1:4, 1:8), (**c**) LC:PMDA (1:2, 1:4, 1:8), (**d**) LC:CA (1:2, 1:4, 1:8), (**e**) GLU2:PMDA (1:2, 1:4, 1:8), and (**f**) GLU2:CA (1:2, 1:4, 1:8), plotted against PMDA/CA-based D-NS (*x*-axis) in deionized water (H_2_O), simulated gastric fluid (pH 1.2), and simulated intestinal fluids (pH 6.8 and 7.4).

Water absorption capacity (WAC), also referred to as swelling capacity (S), significantly influences the surface properties, mobility, and mechanical characteristics of the polymer network, as well as the diffusion of solutes through it. Swelling behavior is particularly important for the application of nanocarriers in drug delivery, making it a favorable property for oral administration. PMDA-based D-NS (Figure 1 and Table 1) exhibits greater swelling capacity than CA-based D-NS due to the higher number of ionizable groups in its structure. This property can be leveraged to enhance the stability, solubility, and bioavailability of poorly water-soluble drugs, as well as to control their release. The high encapsulation efficiency and slow-release kinetics are attributed either to electrostatic interactions between the carboxylic groups of the dianhydride bridges and polar moieties of hydrophilic drugs or to the formation of inclusion complexes with lipophilic drugs. The combination of dextrin and cross-linking agent determines the formation of nanochannels, the degree of hydrogel swelling, drug loading capacity, and drug release rate. Figure 1 illustrates how formulation parameters such as pH, type of building block, and cross-linking agent content affect WAC or S. Increasing the relative amount of cross-linking agent (stoichiometric ratios of 2, 4, and 8) decreases WAC or S because higher cross-linking density restricts polymer chain mobility, creating a more compact structure that limits water diffusion and reduces gel swelling. Figure 1 also shows that the highest swelling occurs in water, and that D-NS exhibits pH-sensitive behavior due to its functional groups. As the pH shifts from neutral to acidic or basic, the swelling percentage decreases. In acidic conditions, –COO– groups convert to –COOH, enhancing hydrogen bonding among hydrophilic groups and increasing physical cross-linking, which reduces swelling. Conversely, at physiological pH (7.4), hydrogen bonding is disrupted, electrostatic repulsion among polymer chains increases, and the hydrogels swell more [44].

### 2.3. Cross-Linking Density and Network Properties

**Figure 2:**
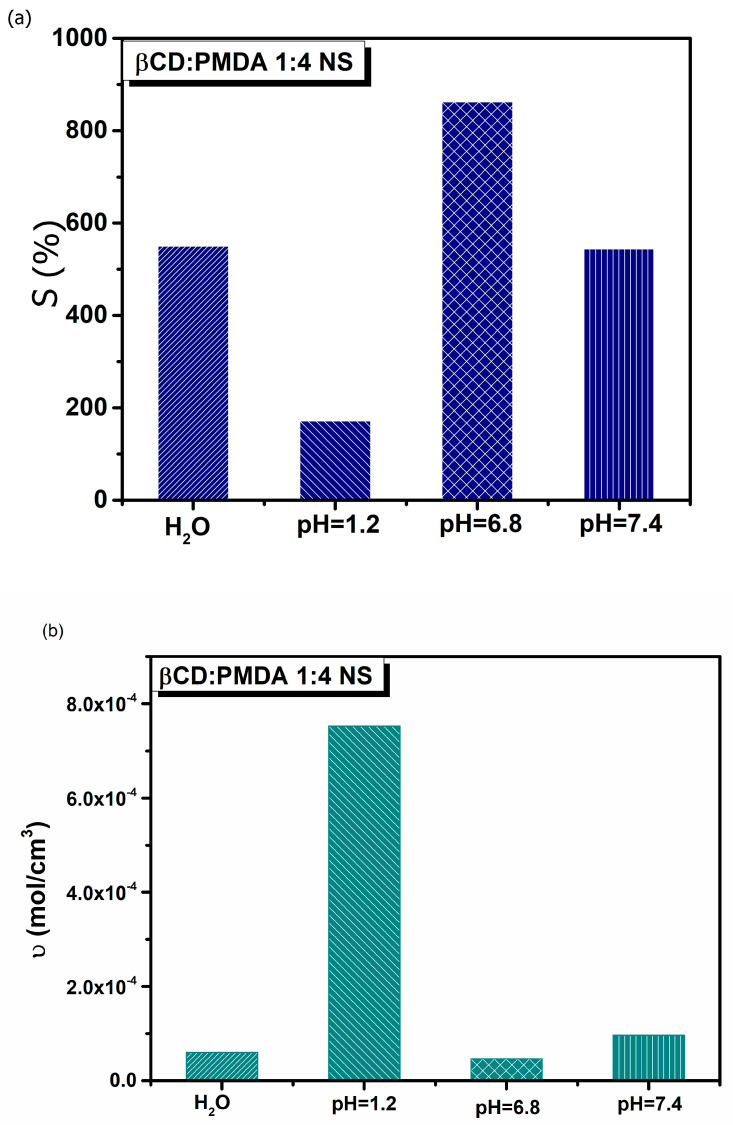
(**a**) Swelling (S, %) and (**b**) cross-linking density (υ, mol/cm^3^) of the β-CD:PMDA 1:4 NS, in deionized water (H_2_O), simulated gastric fluid (pH 1.2), and simulated intestinal fluids (pH 6.8 and 7.4).

The nanosponge (β-CD:PMDA 1:4 NS) was selected for drug studies due to its optimal balance between structural stability and swelling capacity. Our previous study [33] investigated the swelling and cross-linking density of this polymer in detail, providing the basis for exploring additional parameters in the present work. This polymer is also well known for its high drug-loading and encapsulation efficiency. The carboxylate side chains within the network are repelled by ions in the surrounding solution, leading to polymer expansion as the network minimizes charge interactions. As shown in Figure 2a and Table 1, the swelling potential of the polymer decreases under acidic conditions, such as those found in simulated gastric fluid, demonstrating that a pH-responsive swollen polymer system was successfully developed. This system can serve as a versatile oral delivery platform capable of encapsulating and protecting gastric-sensitive bioactives. Moreover, Figure 2b and Table 2 indicate that the cross-links within the polymer matrix are more stable at lower pH values, allowing the polymer chains to extend without detachment from the network. In contrast, at higher pH levels, the cross-links are more susceptible to rupture as the chains stretch, resulting in increased polymer diffusion into the surrounding solution [45].

Controlling the rheological properties of polymeric formulations provides valuable insights into their physical characteristics, structure, stability, and drug release behavior. The rheological properties of candidate polymeric platforms for oral drug delivery were investigated using a TA Instruments Discovery HR 1 Rheometer equipped with a 20 mm diameter stainless steel plate geometry. Two key parameters for characterizing viscoelastic materials are the storage modulus (G′) and the loss modulus (G″), which were measured as a function of frequency in the Frequency Sweep test. In Figures S6 and S7, G′ represents the elasticity of the polymers, while G″ reflects their viscous behavior.

The gel-state behavior of the nanosponges is confirmed by consistently higher G′ values compared to G″ across all angular frequencies (Figures S6 and S7). These analyses also demonstrate that the hydrogels are pH-sensitive. The strength and elasticity of the gel, and consequently the release profile of the active ingredient, can be effectively tuned by selecting appropriate composition and production parameters. As noted previously, swelling is greater at basic pH than under acidic conditions. At higher pH, disruption of hydrogen bonding and ionization of COOH groups lead to increased swelling, which reduces elasticity. Figure S8 shows that nanocarriers with the highest swelling capacities, β-CD: PMDA 1:4 NS (483%) and GLU2:PMDA 1:4 NS (578%), exhibit lower elastic behavior compared to the other formulations, as presented in Table 1.

Furthermore, Figure S9 shows that increasing the amount of cross-linking agent in the polymer network is associated with enhanced elastic behavior.

The cross-linking reaction is an effective strategy for controlling hydrogel properties, as already detailed in our previous study [33], thereby influencing drug release rates. Swelling and mechanical characteristics are key factors in mucoadhesion. Due to the presence of carboxyl and hydroxyl groups, D-NS are expected to be promising candidates for designing mucoadhesive drug delivery systems, as they can prolong the residence time of the formulation on the mucosal surface [46,47].

### 2.4. Mucoadhesive Behavior and Polymer–Mucin Interactions

**Figure 3:**
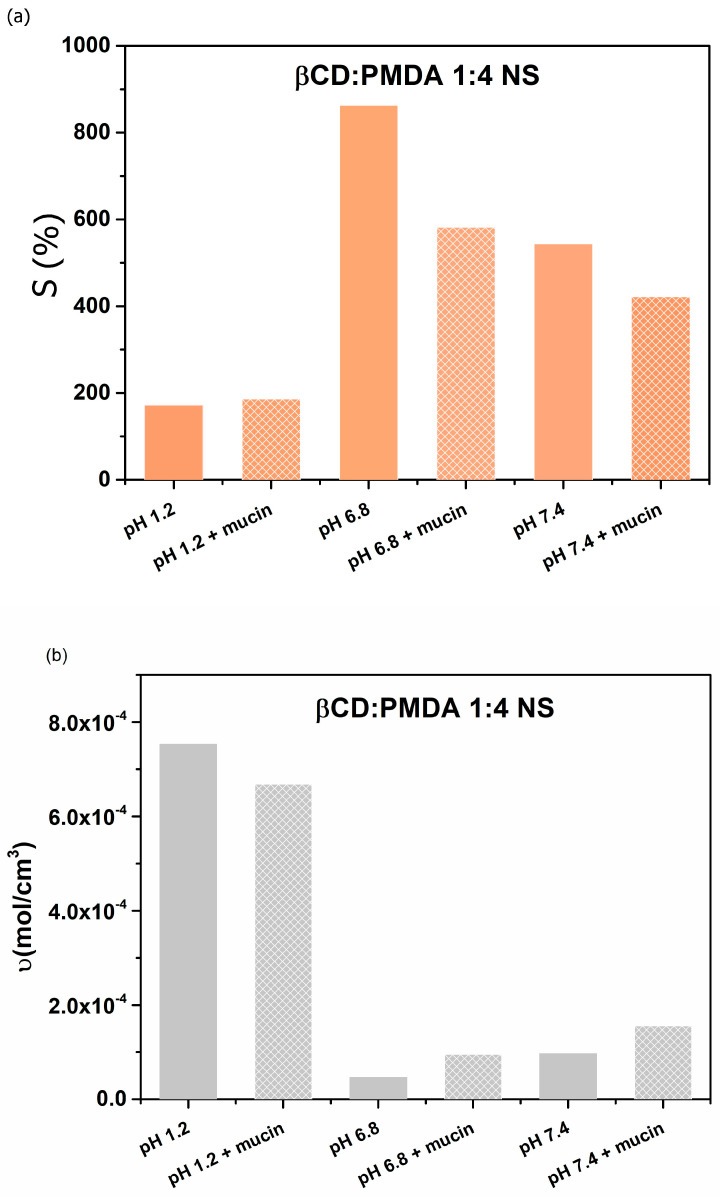
(**a**) Swelling (S, %) and (**b**) cross-linking density (υ, mol/cm^3^) of the β-CD: PMDA 1:4 NS and β-CD:PMDA 1:4 NS + mucin, in simulated fluids: gastric fluid (pH 1.2) and intestinal fluids (pH 6.8 and 7.4).

As shown in Figure 3a, the presence of mucin reduces the swelling of the hydrogel in simulated intestinal fluids (pH 6.8 and 7.4). This effect occurs because mucin forms a protective, gel-like barrier that acts as both a physical and chemical shield against toxins, pathogens, and other irritants. Such behavior underscores the importance of understanding mucus properties, which can be leveraged to enhance drug delivery and help prevent intestinal infections [48]. The maximum swelling is observed at pH 6.8, likely because the polyanionic functional groups can disrupt hydrogen bonds between mucin polymers and compete for hydrogen-bonding sites with mucin glycoproteins, thereby weakening intra- and inter-network cross-links (Figure 3b) and interactions [49]. This behavior can be attributed to the formation of a nanoparticle–mucin network, in which interactions between nanoparticles and mucin chains lead to a more compact gel structure. Such network formation restricts mucin hydration and expansion, resulting in reduced swelling (Figure 3a), and enhanced cross-linking density (Figure 3b) in agreement with observations reported in the literature [50].

**Figure 4:**
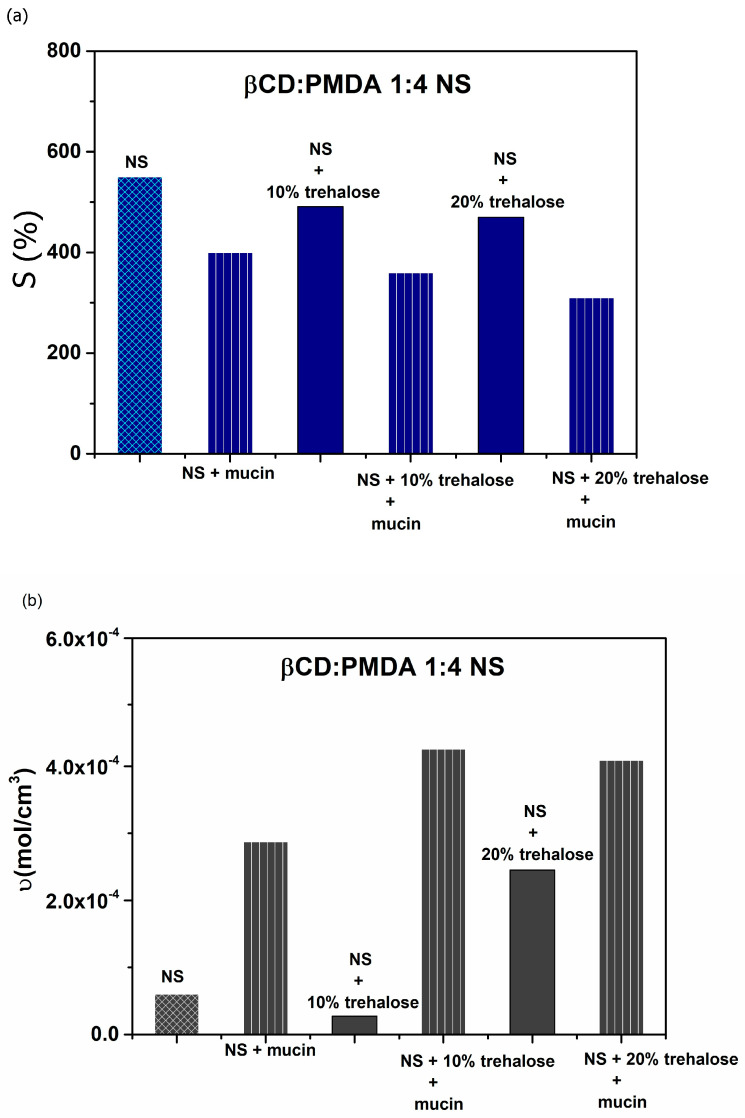
Influence of mucin, 10% trehalose + mucin, and 20% trehalose + mucin on β-CD:PMDA 1:4 NS: (**a**) swelling (S, %) and (**b**) cross-linking density (υ, mol/cm^3^).

Furthermore, 10% and 20% trehalose can be employed as cryoprotectants to prevent particle aggregation of dextrin-based nanosponges, since stability is a key parameter for their use as drug delivery systems. As shown in Figure 4a,b, trehalose at both concentrations did not significantly affect the swelling behavior but influenced the cross-linking density as its concentration varied.

**Figure 5:**
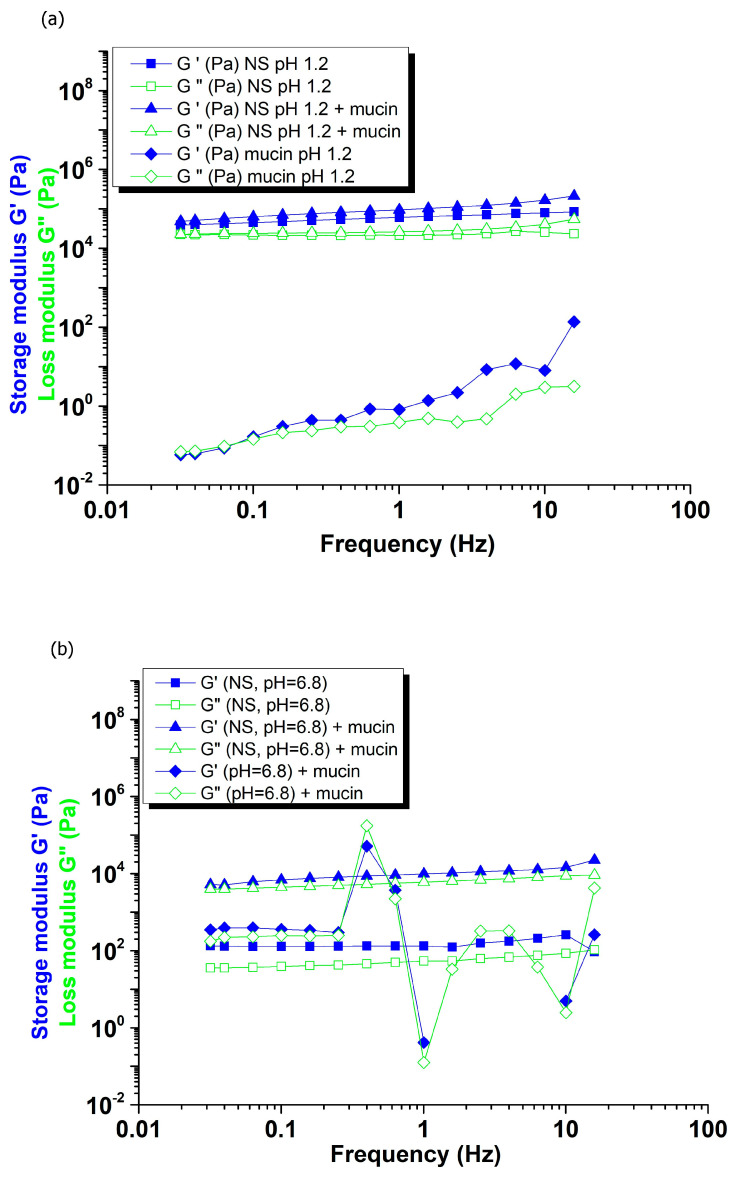

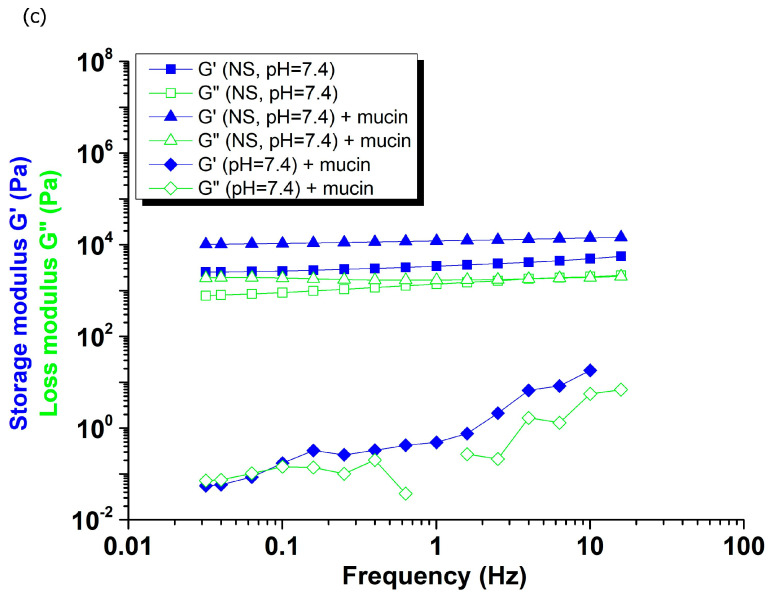
Frequency-dependent storage modulus (G′) and loss modulus (G″) of the β-CD:PMDA (1:4) NS, β-CD:PMDA 1:4 NS + mucin, and mucin, in simulated fluids: (**a**) gastric fluid (pH 1.2), (**b**) intestinal fluid (pH 6.8), and (**c**) intestinal fluid (pH 7.4).

As shown in Figure 5, the viscoelastic moduli (G′ and G″) of the β-CD: PMDA 1:4 NS + mucin mixture increased in both (a) simulated gastric fluid, and (b), (c) simulated intestinal fluids, compared to the individual polymer and mucin solutions. The formation of a cross-linked gel network is confirmed by its rheological behavior, which exhibits minimal frequency dependence, with G′ and G″ remaining nearly constant across a wide frequency range.

**Figure 6:**
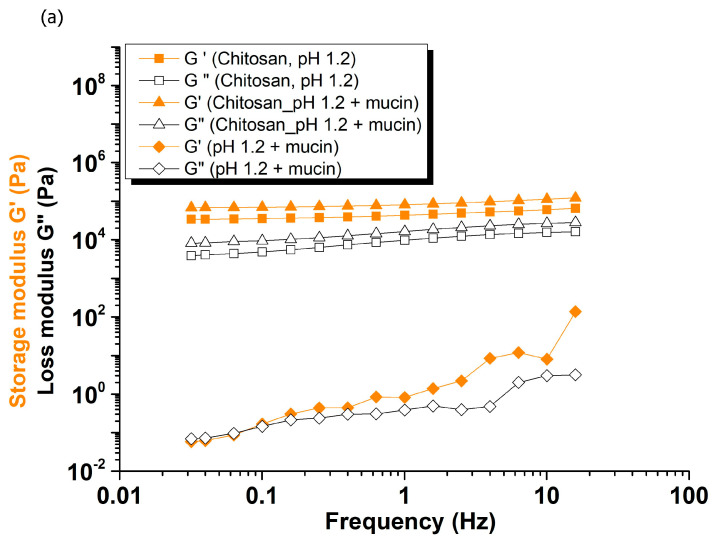

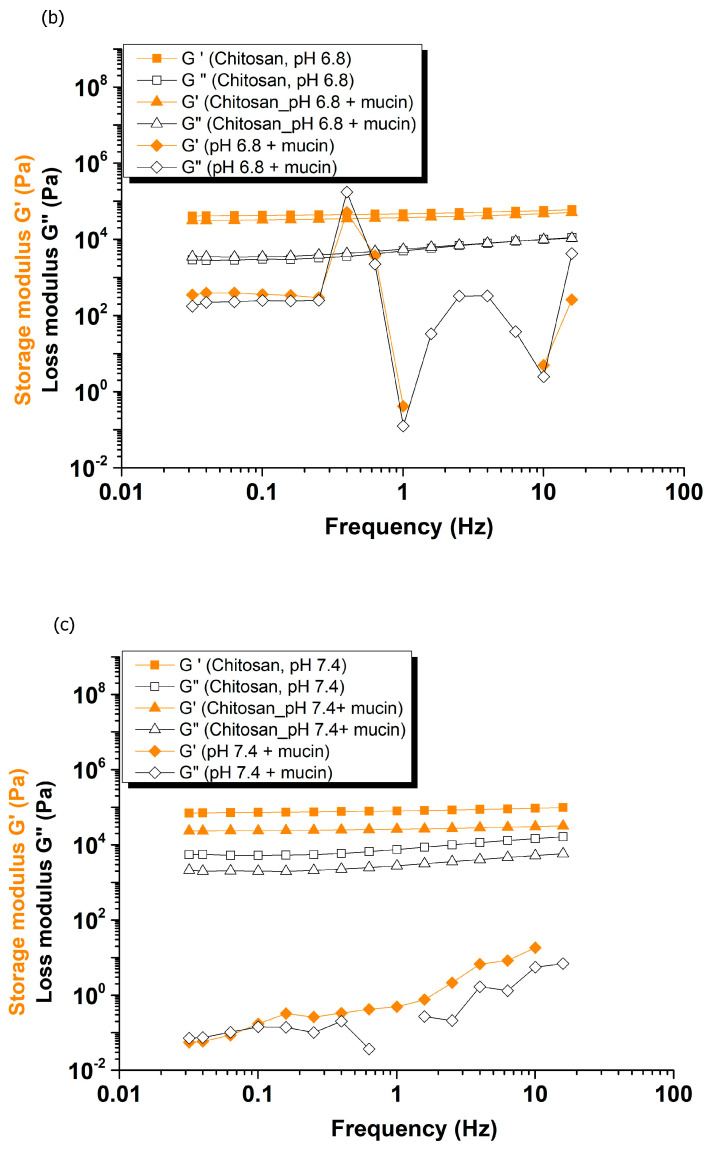
Frequency-dependent storage modulus (G′) and loss modulus (G″) of chitosan, chitosan + mucin, and mucin, in simulated fluids: (**a**) gastric fluid (pH 1.2), (**b**) intestinal fluid (pH 6.8), and (**c**) intestinal fluid (pH 7.4).

Figure 6a shows that both G′ and G″ values are higher for the chitosan–mucin mixture in simulated gastric fluid (pH 1.2) compared to chitosan and mucin alone, as well as to the mixtures in simulated intestinal fluids (pH 6.8 and 7.4) presented in Figure 6b,c [51]. This observation is consistent with literature reports indicating that electrostatic complexation between chitosan and mucin is favored under acidic to mildly acidic conditions (pH 2.4–6.3) [52].

**Figure 7:**
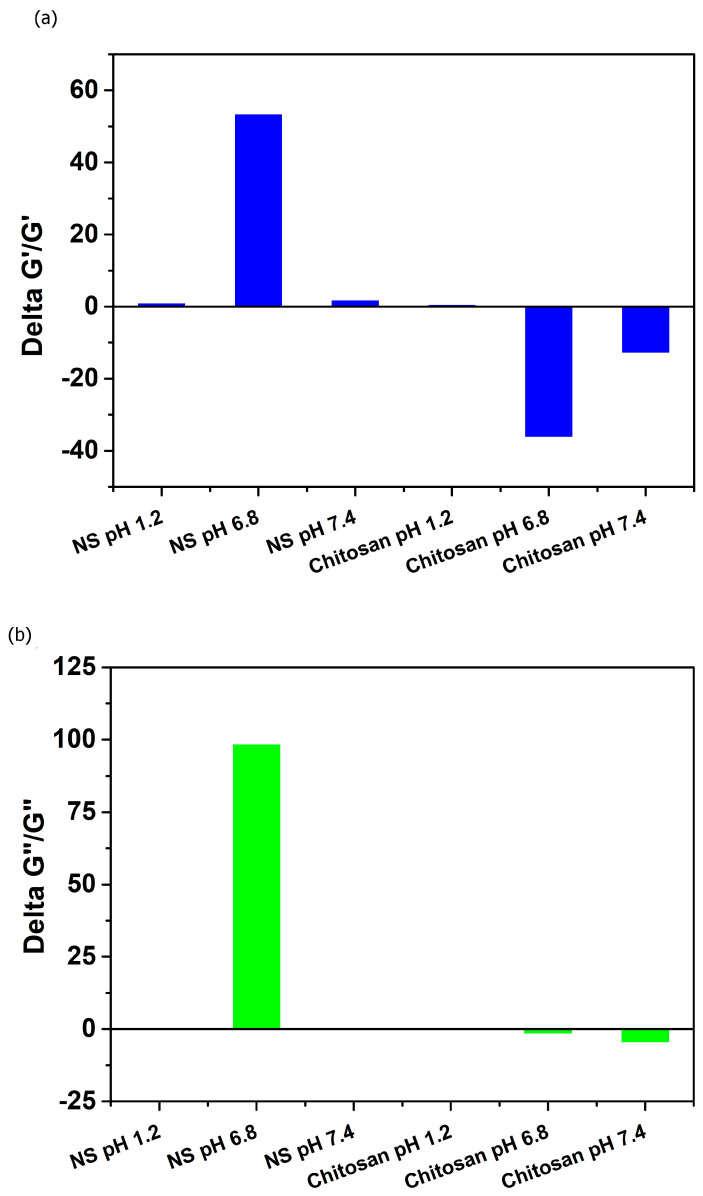
Relative rheological synergism at 10 Hz, expressed as (**a**) ΔG′/G′ and (**b**) ΔG″/G″, for β-CD:PMDA 1:4 (NS) and chitosan, in simulated fluids: gastric fluid (pH 1.2) and intestinal fluids (pH 6.8 and 7.4).

Rheological synergism has been suggested as an effective in vitro parameter for evaluating the mucoadhesive properties of polymers. A higher rheological synergism value indicates a stronger interaction between the polymer and mucin [53]. The mixtures of β-CD: PMDA 1:4 NS and mucin exhibited positive ΔG′/G′ (Figure 7a) and ΔG″/G″ values (Figure 7b), indicating favorable polymer–mucin interactions. Among all tested conditions, the β-CD: PMDA 1:4 NS –mucin mixture at pH 6.8 showed the highest positive rheological synergism, suggesting stronger mucoadhesive behavior. In contrast, negative rheological synergism values were observed for the chitosan–mucin mixtures at pH 6.8 and 7.4, confirming previous literature reports that chitosan is mucoadhesive only within a limited pH range and remains soluble primarily under acidic conditions (pH < 6). At higher pH values, chitosan tends to precipitate, which can compromise the performance of carrier systems [23,54]. At pH 6.8, the high and positive ΔG′ value further supports strong mucin–polymer interactions, leading to the formation of a continuous, swollen, and homogeneous network. This behavior contrasts with that at pH 1.2, where more compact and segregated complexes were formed, limiting elastic response. A low or negative ΔG′ reflects weak interactions between mucin and polymer [55]. Overall, these findings highlight the potential of β-CD: PMDA 1:4 NS as a polymer with promising mucoadhesive capabilities.

**Figure 8:**
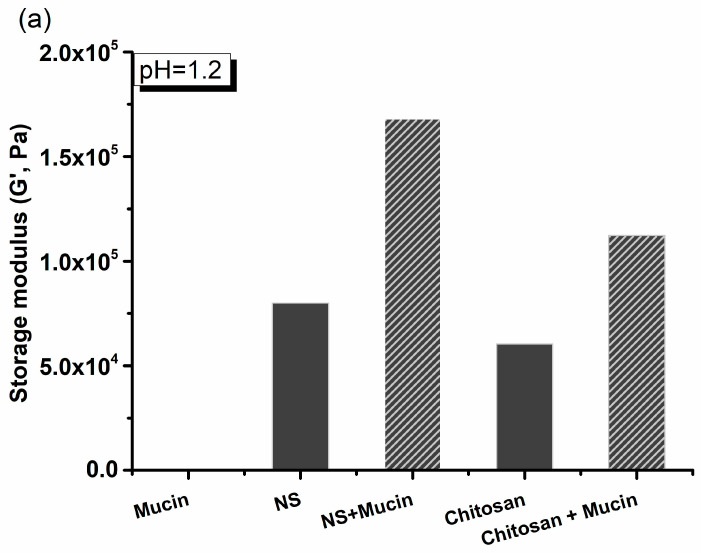

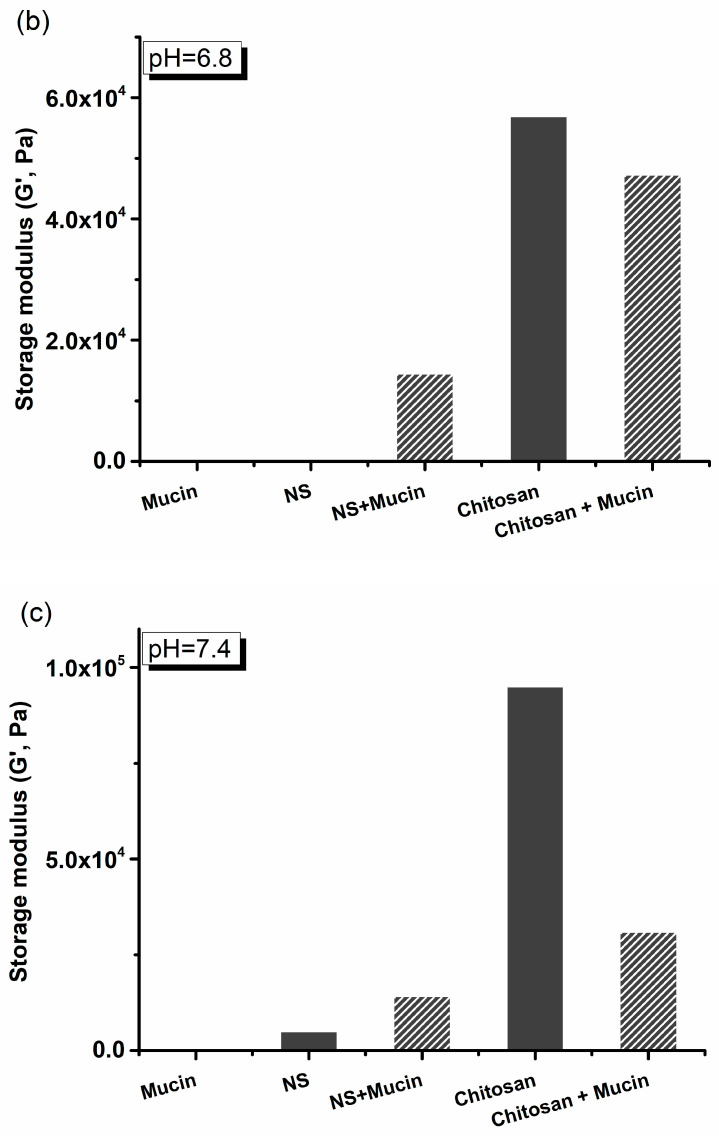
Rheological properties of the β-CD: PMDA 1:4 NS and chitosan, in simulated fluids at 10 Hz: (**a**) gastric fluid (pH 1.2), and (**b**,**c**) intestinal fluids (pH 6.8 and 7.4).

Figure 8 and Table 3 show that polymer–mucus G′ values at 10 Hz are highest in samples swollen in simulated gastric fluid (pH 1.2, Figure 8a). The most pronounced increase in synergistic interactions, as previously observed, was detected with β-CD: PMDA 1:4 NS. At low pH, carboxylic acid groups in the polymer can form hydrogen bonds, resulting in labile intermolecular cross-links. As the pH increases (Figure 8b,c), these hydrogen bonds are replaced by ionic interactions, leading to disruption of the hydrogen-bonded network. Consequently, mucus–polymer mixtures lose structural integrity and progressively exhibit more viscous behavior. Interactions between mucus and mucoadhesive polymers are primarily mediated by secondary bonding, mainly hydrogen bonding, and physical entanglement. A strengthened gel network can form spontaneously when the polymer is overhydrated, as the presence of excess water facilitates additional network links. Fully hydrated mucus gels allow more interactions between polymer chains and mucin glycoproteins, contributing to a stronger network. Polymers with a high density of hydrogen-bonding groups can therefore interact more effectively with mucin. Previous studies have suggested that polymer concentration can influence the extent of rheological synergism, which will be explored in our future work [56,57].

### 2.5. Apomorphine (APO)-Loaded D-NS Formulations

βCD, GLU_2,_ and LC-based NS showed the capability to load apomorphine (APO) with good encapsulation efficiency (75.5–90.6%) and loading capacity ranging from 6.86 to 8.23% (Table 4).

The addition of antioxidant agents (sodium metabisulfite, ascorbic acid) to the NS suspension did not affect the NS loading capacity. To reduce D-NS size and obtain nanosuspensions suitable for drug loading, D-NS (at a 1:4 molar ratio of dextrin to cross-linking agent) were processed using ball milling or high-pressure homogenization (HPH), considering the different characteristics of each polymer matrix. Stable nanosuspensions of GLU_2_:PMDA NS, GLU_2_:CA NS, and LC:CA NS were obtained by milling the D-NS powders, followed by suspension in water. In contrast, β-CD:PMDA NS, β-CD:CA NS, and LC:PMDA NS required high-pressure homogenization to achieve stable nanosuspensions.

The average diameters of all the D-NS (Table 5) were reduced after top-down methods (ballmill or HPH), reaching values between 178 and 442 nm. All resulting nanosuspensions exhibited favorable polydispersity index values (0.11–0.30), indicating a more homogeneous size distribution compared to the coarse powders. Table 6 reports the physico-chemical parameters of NS formulations after loading with the drug, showing a slight increase in size and a decrease in zeta potential values. Understanding the zeta potential in nanoparticle preparation aids in predicting the nanoparticles’ in vitro and in vivo behavior and evaluating the stability of colloidal systems. Changes in zeta potential and particle size properties have significant biological implications for cellular internalization, pharmacokinetics, and biodistribution [58]. The surface charge on particles significantly influences the stability of the nanoparticle formulation, as strong electrostatic repulsion among particles can enhance stability. The zeta potential measurements reveal that PMDA and CA introduce an anionic nature to the synthesized nanocarriers due to the presence of carboxylic acid groups (-COOH), which primarily gain charge through deprotonation until equilibrium is achieved. The zeta potential values of PMDA and CA-based D-NS ranged from −16.00 mV to −32.60 mV. The zeta potential values, which indicate the surface properties of the nanosized products, confirmed the stability of the suspensions.

**Figure 9:**
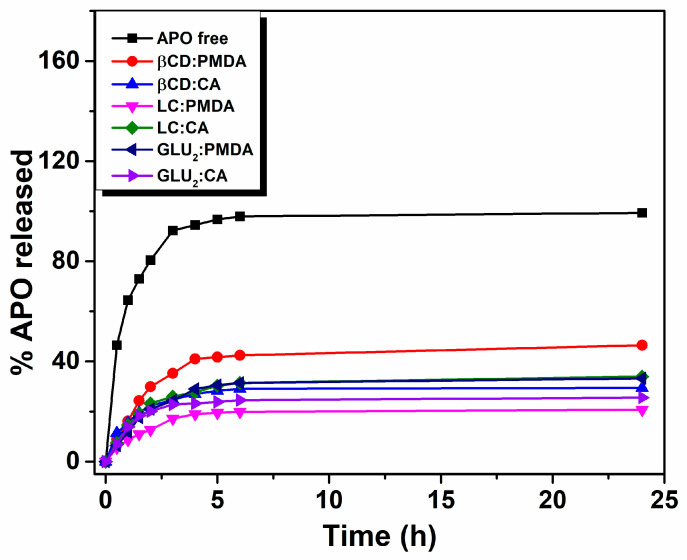
In vitro release profiles of apomorphine (APO) from dextrin-based nanosponges (D-NS) formulations.

Stability studies demonstrated no significant changes in the physicochemical parameters of the three types of D-NS stored at 4 °C for up to 6 months, and no evidence of APO chemical degradation. These findings indicate that incorporation of APO into the D-NS polymeric matrix effectively protects the drug from oxidative degradation. In vitro release studies further revealed a slow and sustained release of APO from D-NS in simulated intestinal fluid, in contrast to the rapid diffusion observed for the free drug (APO) (Figure 9).

## 3. Conclusions

A series of dextrin-based nanosponges (D-NS) was successfully synthesized using β-CD, GLU_2_, and LC as building blocks, with pyromellitic dianhydride (PMDA) and citric acid (CA) as cross-linking agents at varying stoichiometric ratios (1:2, 1:4, and 1:8). The resulting polymers exhibited pH-responsive and mucoadhesive properties, attributed to their carboxylic and saccharide functional groups, and they demonstrated the ability to form stable hydrogels under physiological conditions. After top-down processing (ball milling or high-pressure homogenization), particle sizes were reduced to 178–442 nm with uniform distributions (PDI 0.11–0.30) and stable surface charges (−16.00 to −32.60 mV). Swelling studies revealed a pH-dependent behavior (simulated gastric fluid, pH 1.2, and simulated intestinal fluids, pH 6.8 and pH 7.4), with reduced swelling under acidic conditions due to enhanced hydrogen bonding and cross-linking, while physiological pH promoted higher expansion through electrostatic repulsion. The presence of mucin further decreased swelling, confirming polymer–mucin interactions. Rheological synergism analysis identified β-CD:PMDA 1:4 NS as the most mucoadhesive formulation, showing the highest positive ΔG′/G′ values at pH 6.8. β-CD-, GLU_2_-, and LC-based nanosponge (NS) showed high apomorphine (APO) loading (6.86–8.23%) and encapsulation efficiency (75.5–90.6%). Stability studies at 4 °C for six months revealed no changes in physicochemical properties or APO degradation, indicating effective protection by the polymer matrix. In vitro release studies demonstrated slow, sustained APO release from D-NS compared to rapid diffusion of the free drug. These findings highlight the potential of β-CD:PMDA 1:4 NS as a promising mucoadhesive carrier for oral drug delivery. Its pH sensitivity, controlled swelling, and strong interaction with mucin suggest suitability for sustained release applications targeting the gastrointestinal tract. Future studies will focus on drug loading efficiency, in vivo pharmacokinetics, and bioavailability to validate the effectiveness of β-CD:PMDA 1:4 NS and other D-NS formulations for oral administration. In addition, more detailed investigations will be conducted on these pH-sensitive, mucoadhesive polymers to assess their potential in neurodegenerative disease therapy.

## 4. Materials and Methods

### 4.1. Materials

β-cyclodextrin (β-CD, Mw = 1134.98 g/mol), GluciDex^®^2 (GLU_2_, DE value of 2; Mw = ~200,000 g/mol), and KLEPTOSE^®^ Linecaps (LC, Mw = ~12,000 g/mol) (kindly supplied as a gift by Roquette, Lestrem, France), are used as building blocks, whereas pyromellitic dianhydride (PMDA) and citric acid (CA) as multifunctional cross-linking agents. Anhydrous β-CD, LC, and GLU_2_ were kept in an oven prior to use. Pyromellitic dianhydride (PMDA, 97.00%); dimethylsulfoxide (DMSO, ≥99.90%); triethylamine (Et_3_N, ≥99.00%); acetone (C_3_H_6_O, ≥99.00% (GC)), sodium hypophosphite monohydrate (NaPO_2_H_2_*H_2_O, ≥99.00%), hydrochloric acid (HCl, 37.00%); sodium hydroxide (NaOH, pellets); mucin (from porcine stomach), chitosan (low molecular weight), sodium chloride (NaCl, ACS, ISO, Reag. Ph Eur), potassium chloride (KCl, ≥99.50% (AT)), are all purchased from Sigma-Aldrich (Darmstadt, Germany). The citric acid (C_6_H_8_O_7_, 99.90%) is purchased from VWR Chemicals BDH (Milano, Italy). Disodium hydrogen phosphate dodecahydrate (Na_2_HPO_4_*2H_2_O, 99.00%) and potassium phosphate monobasic (KH_2_PO_4_, 98.00%) are purchased from Italia Carlo Erba S. P. A (Milano, Italy). Simulated gastric fluid (SGF, pH 1.2) is prepared by dissolving 1.00 g of NaCl and adding 3.50 mL of concentrated HCl, then diluting the solution to 500 mL with deionized water. Simulated intestinal fluid (SIF, pH 6.8) is prepared by mixing 6.80 g of KH_2_PO_4_ in 250 mL of water with 0.94 g of NaOH dissolved in 118 mL of water, and then diluting the mixture to a final volume of 500 mL. Simulated intestinal fluid (SIF, pH 7.4) is prepared by dissolving 4.00 g of NaCl, 0.10 g of KCl, 0.90 g of Na_2_HPO_4_·2H_2_O, and 0.12 g of KH_2_PO_4_ in 500 mL of deionized water. Deionized water and water purified by reverse osmosis (MilliQ water, Millipore, Burlington, MA, USA) with a resistivity above 18.20 MΩcm^−1^, and dispensed through a 0.22 μm membrane filter, are used throughout the studies.

### 4.2. Synthesis of Dextrin-Based Polymers

Dextrin-based nanosponges (D-NS) are chemically cross-linked polymers formed through the reaction of dextrin building blocks with a suitable cross-linking agent under defined conditions (Figure S1 in Supplementary Materials).

#### 4.2.1. Synthesis of PMDA-Based D-NS

The synthesis was performed as previously described [33]. Initially, 4.89 g of anhydrous β-CD, GLU_2_, or LC was dissolved in 20 mL of dimethyl sulfoxide (DMSO, ≥99.9%) in a round-bottom flask. After obtaining a homogeneous solution, 2.5 mL of triethylamine (Et_3_N, ≥99%) was added to catalyze the reaction, followed by the addition of pyromellitic dianhydride (PMDA, 97%) as the cross-linking agent in molar ratios of 2, 4, or 8 per glucose unit (Table 7). The polymerization reaction occurred rapidly at room temperature, and after 24 h, the resulting solid product was collected and purified by Büchner filtration using Whatman No. 1 filter paper (Whatman, Maidstone, UK). The by-products were subsequently removed by Soxhlet extraction with acetone for approximately 48 h. Finally, a homogeneous white powder of PMDA-based D-NS was obtained, with a yield exceeding 95%.

#### 4.2.2. Synthesis of CA-Based D-NS

The synthesis was performed as previously described [43]. The nanosponge was synthesized by dissolving 4.40 g of anhydrous β-CD, GLU_2_, or LC in 15 mL of deionized water, followed by the addition of 0.80 g sodium hypophosphite monohydrate (SHP, ≥99%) as a catalyst and citric acid (CA, 99.9%) as the cross-linking agent at molar ratios of 2, 4, or 8 per glucose unit (Table 7). The reaction was carried out under vacuum in an oven at 140 °C and 100 °C until a solid, insoluble mass was formed. The resulting solid was purified by Büchner filtration with deionized water and acetone to eliminate by-products. Ultimately, a homogeneous white powder of CA-based D-NS was obtained, with a yield of 60%.

### 4.3. Characterization

The prepared polymers were analyzed by the following techniques:

Fourier-Transform Infrared Spectroscopy (FTIR) Analysis—by a Perkin Elmer Spectrum Spotlight 100 FTIR spectrophotometer equipped with Spectrum software (Application Version: 10.03.05.0099) (PerkinElmer, Waltham, MA, USA). The FTIR spectra were collected in the spectral range of 4000–650 cm^−1^, at a spectral resolution of 4 cm^−1^, and 8 scans per sample/background. The measurements were performed using a versatile Attenuated Total Reflectance (FTIR-ATR) sampling accessory equipped with a diamond crystal plate.

Thermogravimetric Analysis (TGA)—using a TA Instrument Thermogravimetric Analyzer (TGA), Q500 (TA Instruments, New Castle, DE, USA). About 10 mg of each sample was placed in an aluminum pan and heated from room temperature to 800 °C at a ramp rate of 10 °C/min under a nitrogen (N_2_) atmosphere. Gas flow rates of 40 mL/min in the balance section and 60 mL/min in the furnace section were applied.

Zeta Potential and Particle Size Analyses—Dynamic light scattering (DLS) measurements were performed using a Malvern Zetasizer Nano ZS, with data acquisition and analysis conducted through DTS Version 5.03 software (Malvern Instruments Ltd., Worcestershire, UK). Approximately 1 mg of the sample was dispersed in 1 mL of Milli-Q water, and the average particle sizes were reported as intensity-weighted distributions based on hydrodynamic diameters (d_H_).

### 4.4. Swelling Studies

The swelling kinetics of the synthesized nanosponges were evaluated by monitoring their weight gain upon immersion in deionized water and in aqueous media at pH 1.2, 6.8, and 7.4. For these measurements, 0.3 g of dry powder was immersed in deionized water, simulated gastric fluid (pH 1.2), and simulated intestinal fluids (pH 6.8 and 7.4) in 15 mL test tubes. The samples were initially homogenized using a vortex mixer, after which the test tubes were sealed and maintained at room temperature. Upon reaching equilibrium swelling, the mixtures were centrifuged to separate the water-bound material from the unabsorbed free water. The supernatant was carefully removed, and any residual free water was gently blotted with tissue paper prior to recording the sample weight. The swelling percentage (%S) was calculated according to Equation (1):

(1) $$S\;(\%)\;=\;\frac{\left(m_{s}-m_{o}\right)}{m_{o}}\times \;100$$

where $m_{s}$ is the weight of the swollen sample, and $m_{o}$ is the initial weight of the dry sample.

### 4.5. Preparation of Mucin Samples

To investigate the dominant interactions between mucin and the polymers, mucin suspensions were prepared by dispersing 250 mg of mucin and 500 mg of polymer in 10 mL of deionized water, and at both simulated gastric fluid (pH 1.2), and simulated intestinal fluid (pH 6.8 and pH 7.4) in 15 mL test tubes. The mixtures were briefly vortexed to ensure homogeneity and incubated at room temperature for 2 h to allow hydration and swelling. After 2 h, the samples were centrifuged, and the supernatant was carefully removed. To investigate the role of trehalose in modulating particle aggregation and its pH-dependent effects on swelling behavior, cross-linking density, and polymer–mucin interactions, trehalose was incorporated at concentrations of 10% and 20% (*w*/*v*). Chitosan was employed as a reference standard to benchmark and compare the mucoadhesive capacity of the synthesized nanosponges. Chitosan was utilized as a control to evaluate mucoadhesive performance.

### 4.6. Cross-Linking Density Determination

The previously prepared equilibrium-swollen samples were used to determine the polymer volume fraction (υ_2m_), which, in turn, enabled the determination of the cross-linking density (υ) according to Flory–Rehner theory. The cross-linking density, defined as the number of cross-links per unit volume in a polymer network, was calculated using Equation (2), as described in our earlier publication [33].

(2)    υmolcm3=−[ln1−υ2m+υ2m+λ1 (υ2m2]V1 [υ2m ^1/3 −(2f υ2m )]

### 4.7. Rheological Analysis

Rheological measurements were performed using a TA Instruments Discovery HR Rheometer, equipped with a 20 mm stainless steel plate geometry and Peltier-controlled temperature regulation. Frequency sweep measurements were carried out over a range of 0.2 to 100 rad/s with a 2% stress amplitude, collecting 5 points per decade. Oscillatory shear mode was employed to evaluate the viscoelastic properties, specifically the storage modulus (G′) and loss modulus (G″), of the swollen nanosponges as a function of frequency. The swollen sample was positioned between the upper parallel plate and the stationary surface with a 0.5 mm gap and equilibrated at 25 °C for 300 s prior to measurements, in accordance with the established protocol [33].

### 4.8. Mucoadhesion Studies

Rheological synergism parameters, representing the deviation between the measured viscoelastic behavior of the mucin–polymer mixtures and the theoretical sum of polymer and mucin components, were calculated according to the literature (Equations (3a) and (3b)) [57,59]:

(3a) $$\;\;∆G^{\prime }={G^{\prime }}_{\left(mix\right)}-({G^{\prime }}_{\left(polymer\right)}+{G^{\prime }}_{\left(mucin\right)})$$

(3b) $$∆G^{\prime \prime }={G^{\prime \prime }}_{\left(mix\right)}-({G^{\prime \prime }}_{\left(polymer\right)}+{G^{\prime \prime }}_{\left(mucin\right)})$$

The relative rheological synergism, which quantifies the increase in viscoelastic response of the mucin–polymer mixture relative to the separate components, was calculated as described in the literature (Equations (4a) and (4b)).

(4a) $$\Delta G^{\prime }/G^{\prime },\;\mathrm{where}\;G^{\prime }={G^{\prime }}_{\left(polymer\right)}+{G^{\prime }}_{\left(mucin\right)}$$

(4b) $$\Delta G^{\prime \prime }/G^{\prime \prime },\;\mathrm{where}\;G^{\prime \prime }={G^{\prime \prime }}_{\left(polymer\right)}+{G^{\prime \prime }}_{\left(mucin\right)}$$

For the calculation of rheological synergism, the G′ and G″ moduli recorded at a frequency of 10.04 Hz were utilized.

### 4.9. Preparation of D-NS Nanosuspensions

Aqueous nanosuspensions of the synthesized D-NS (β-CD:PMDA, β-CD:CA, LC:PMDA, LC:CA, GLU_2_:PMDA, GLU_2_:CA) were prepared and subjected todifferent top-down processing methods (ball milling, high-shear homogenizer, and high-pressure homogenizer), to reduce NS size and obtain a more homogenous nanoparticle distribution. Ball milling was applied to the D-NS coarse powder. For high-pressure homogenization (HPH), the D-NS were first suspended in water at a concentration of 10 mg/mL and pre-homogenized using a high-shear homogenizer (Ultra-Turrax, IKA-Werke, Staufen, Germany) for 10 min at 24,000 rpm. The resulting suspension was then subjected to size reduction by high-pressure homogenization using an EmulsiFlex C5 Avastin homogenizer (Avestin Inc., Ottawa, ON, Canada) for 90 min at a back pressure of 500 bar.

### 4.10. Incorporation into D-NS Nanocarriers

Apomorphine (APO) was incorporated into the prepared D-NS nanosuspensions (10 mg/mL) by incubation under continuous stirring overnight at room temperature and protected from light. A drug concentration of 1 mg/mL was evaluated in the presence of various antioxidants, including 0.1% ascorbic acid, 0.05% EDTA, and 0.1% sodium meta bisulfite. HPLC analysis was used to quantify the loaded drug, enabling calculation of encapsulation efficiency and loading capacity. In vitro chemical and physical stability, along with drug release kinetics, were further assessed. In vitro APO release studies were conducted using a dialysis bag (cellulose membrane, 12 kDa cut-off, Spectra/Pore) with simulated intestinal fluid (pH 6.8) as the receiving medium.

### 4.11. Characterization of APO-Loaded Formulations

APO content in nanosponges was quantified using an Agilent 1100 HPLC system with a fluorimetric detector (λ_ex = 270 nm, λ_em = 450 nm). Separation was performed on a 250 × 4.0 mm, 5 μm Nucleosil 100-C18 column (Agilent, Santa Clara, CA, USA) at 1 mL/min. The mobile phase consisted of methanol and 0.1 M phosphate buffer (pH 3, 30:70) with 20 mg/L sodium octanesulfonate and 10 mg/L Na_2_EDTA. An optimized extraction procedure was applied to recover APO from the loaded nanosponges prior to HPLC analysis.

## Figures and Tables

**Table 1:** Swelling (S, %) of D-NS: experimental values in deionized water (H_2_O) and in solutions with pH 1.2, 6.8, and 7.4.

Samples		Swelling (S, %)		
	H_2_O	pH = 1.2	pH = 6.8	pH = 7.4
GLU_2_:CA 1:2	346	388	357	367
GLU_2_:CA 1:4	229	257	267	273
GLU_2_:CA 1:8	118	118	105	114
LC:CA 1:2	561	534	601	580
LC:CA 1:4	351	375	557	375
LC:CA 1:8	158	136	137	155
β-CD:CA 1:2	235	364	211	301
β-CD:CA 1:4	235	271	210	272
β-CD:CA 1:8	92	192	97	128
β-CD:PMDA 1:2	1348	551	912	861
β-CD:PMDA 1:4	483	211	436	409
β-CD:PMDA 1:8	254	136	224	220
LC:PMDA 1:2	1364	629	942	867
LC:PMDA 1:4	376	209	342	336
LC:PMDA 1:8	151	125	169	158
GLU_2_:PMDA 1:2	741	351	533	521
GLU_2_:PMDA 1:4	578	272	516	445
GLU_2_:PMDA 1:8	259	188	240	242

**Table 2:** β-CD: PMDA 1:4 NS Quantitative values of swelling (S, %) and cross-linking density (υ, mol·cm^−3^) of the β-CD:PMDA 1:4 NS measured in deionized water (H_2_O), in simulated gastric fluid (pH 1.2), and in simulated intestinal fluids (pH 6.8 and 7.4).

Sample (βCD:PMDA 1:4 NS)	S (%)	υ (mol/cm^3^)
Deionized H_2_O	550	6.04 × 10^−5^
Gastric fluid (pH = 1.2)	172	7.53 × 10^−4^
Intestinal fluid (pH = 6.8)	863	4.64 × 10^−5^
Intestinal fluid (pH = 7.4)	544	9.65 × 10^−5^

**Table 3:** Storage modulus (G′, Pa) of the samples in simulated fluids at 10 Hz: gastric fluid (pH 1.2) and intestinal fluids (pH 6.8 and 7.4), including the calculated interaction terms (ΔG′/G′).

Samples	G′ (Pa) (pH = 1.2)	G′ (Pa) (pH = 6.8)	G′ (Pa) (pH = 7.4)	Delta G′/G′ (pH = 1.2)	Delta G′/G′ (pH = 6.8)	Delta G′/G′ (pH = 7.4)
Mucin	8.05	4.95	18.22			
NS	80,009.00	260.29	4940.29			
NS + Mucin	167,957.00	14,444.50	14,132.70	1.09	53.45	1.85
Chitosan	60,420.80	56,871.10	94,889.30			
Chitosan + Mucin	112,273.00	47,256.30	30,918.20	0.64	−36.26	−12.90

**Table 4:** Loading capacity and encapsulation efficiency of apomorphine (APO)-loaded dextrin-based nanosponges (D-NS).

Samples	Loading Capacity (%)	Encapsulation Efficiency (%)
β-CD:CA-APO	8.09 ± 1.05	89.0 ± 1.20
LC:CA-APO	7.11 ± 0.90	78.19 ± 1.30
GLU_2_:CA-APO	6.86 ± 1.40	75.47 ± 2.00
β-CD:PMDA-APO	8.23 ± 1.15	90.58 ± 2.04
LC:PMDA-APO	7.40 ± 1.20	81.41 ± 1.75
GLU_2_:PMDA-APO	7.45 ± 0.65	81.85 ± 1.06

**Table 5:** Physicochemical parameters of D-NS nanosuspensions, including Z-average hydrodynamic diameter (Z-Ave), polydispersity index (PDI), and zeta potential values (mean ± SD).

Samples	Z-Ave ± SD (nm)	PDI	Zeta Potential ± SD (mV)
GLU_2_:CA 1:4	186.60 ± 2.54	0.25 ± 0.02	−34.31 ± 0.78
LC:CA 1:4	298.90 ± 9.08	0.21 ± 0.03	−28.58 ± 0.52
β-CD:CA 1:4	441.70 ± 9.87	0.26 ± 0.02	−22.75 ± 0.43
β-CD:PMDA 1:4	286.60 ± 3.94	0.20 ± 0.01	−30.21 ± 0.75
LC:PMDA 1:4	382.76 ± 13.96	0.30 ± 0.04	−30.16 ± 1.28
GLU_2_:PMDA 1:4	177.80 ± 2.66	0.11 ± 0.01	−22.50 ± 0.76

**Table 6:** Physicochemical characteristics of apomorphine (APO)-loaded dextrin-based nanosponges (D-NS), including particle size, polydispersity index (PdI), and zeta potential values (mean ± SD).

Samples	Particle Size ± SD (nm)	PdI	Zeta Potential ± SD (mV)
β-CD:CA-APO	444.8±22.3	0.19	−19.50±1.81
LC:CA-APO	310.3±20.6	0.22	−22.65±2.85
GLU_2_:CA-APO	225.5±11.7	0.21	−24.54±3.26
β-CD:PMDA-APO	304.5±15.6	0.22	−26.85±2.04
LC:PMDA-APO	402.5±12.4	0.22	−28.04±2.45
GLU_2_:PMDA-APO	202.4±16.9	0.23	−20.45±3.12

**Table 7:** Amounts of cross-linking agents used for the preparation of D-NS at different molar ratios.

Nanosponges	Molar Ratio (Dextrin:Cross-Linking Agent)	Molar Ratio (Glucose Unit: Cross-Linking Agent)	Mass of Cross-Linking Agent (g)
β-CD/LC/GLU_2_:PMDA	1:2	1:0.29	1.87
	1:4	1:0.57	3.75
	1:8	1:1.14	7.51
β-CD/LC/GLU_2_:CA	1:2	1:0.29	1.49
	1:4	1:0.57	2.98
	1:8	1:1.14	5.96

## Data Availability

The original contributions presented in this study are included in the article/Supplementary Materials. Further inquiries can be directed to the corresponding author.

## Supplementary Materials

The following supporting information can be downloaded at: https://www.mdpi.com/article/10.3390/gels12010090/s1, Figure S1: Schematic representation of the synthesis of β-CD: PMDA and β-CD: CA nanosponges (NS); Figure S2: FTIR spectra of PMDA-based D-NS: (a) β-CD: PMDA NS, (b) LC:PMDA NS, (c) Glu_2_:PMDA NS; Figure S3: FTIR spectra of CA-based D-NS: (a) β-CD:CA NS, (b) LC:CA NS, (c) Glu_2_:CA NS; Figure S4: TGA curves of PMDA-based D-NS: (a) β-CD:PMDA NS, (b) LC:PMDA NS, (c) Glu_2_: PMDA NS; Figure S5: TGA curves of CA-based D-NS: (a) β-CD:CA NS, (b) LC:CA NS, (c) Glu_2_:CA NS; Figure S6: Storage (G′) and loss (G″) modulus versus angular frequency for (a) β-CD:PMDA 1:4 NS; (b) LC:PMDA 1:4 NS; and (c) Glu_2_:PMDA 1:4 NS; 0.8 mm gap size. Nanocarriers were allowed to swell in deionized water and in aqueous solutions at pH 1.2, 6.8, and 7.4.; Figure S7: Storage (G′) and loss (G″) modulus versus angular frequency for (a) β-CD:CA 1:4 NS; (b) LC:CA 1:4 NS; and (c) Glu_2_:CA 1:4 NS; 0.8 mm gap size. Nanocarriers were allowed to swell in deionized water and in aqueous solutions at pH 1.2, 6.8, and 7.4.; Figure S8: Storage (G′) and loss (G″) modulus versus angular frequency for β-CD:PMDA 1:4 NS; β-CD:CA 1:4 NS; LC:PMDA 1:4 NS; LC:CA 1:4 NS; Glu_2_:PMDA 1:4 NS; Glu_2_:CA 1:4 NS; 0.8 mm gap size. Nanocarriers are swollen in deionized water (H_2_O); Figure S9: Storage (G′) and loss (G″) modulus versus angular frequency for β-CD:PMDA 1:2 NS; β-CD:PMDA 1:4 NS; and β-CD:PMDA 1:8 NS; 0.8 mm gap size. Nanocarriers are swollen in deionized water (H_2_O).

## Abbreviations

The following abbreviations are used in this manuscript:

β-CD	β-Cyclodextrin
LC	KLEPTOSE^®^ Linecaps
GLU2	GluciDex^®^2
CA	Citric Acid
PMDA	Pyromellitic Dianhydride
GI	Gastrointestinal
CDs	Cyclodextrins
D-NS	Dextrin-based Nanosponges
NS	Nanosponge
AD	Alzheimer’s disease
PD	Parkinson’s disease
DMSO	Dimethylsulfoxide
H_2_O	Deionized water
Et_3_N	Triethylamine
SHP	Sodium Hypophosphite Monohydrate
FTIR	Fourier-Transform Infrared Spectroscopy
TGA	Thermogravimetric Analysis
HPLC	High-Performance Liquid Chromatography
DLS	Dynamic Light Scattering
N_2_	Nitrogen
S	Swelling
WAC	Water Absorption Capacity
G	Shear Modulus
G′	Storage Modulus
G″	Loss Modulus
ΔG′	Rheological Synergism
ΔG′/G′	Relative Rheological Synergism
-COOH	Carboxylic Acid Groups
-OH	Hydroxyl Groups
PDI	Polydispersity Index
Z-Ave	Z-average
HPH	High-Pressure Homogenizer/Homogenization
APO	Apomorphine
BBB	Blood-Brain Barrier
PAA	Poly(acrylic acid)
PVP	Poly(vinyl pyrrolidone)
