# Supplementary material for: pH-Sensitive Dextrin-Based Nanosponges Crosslinked with Pyromellitic Dianhydride and Citric Acid: Swelling, Rheological Behavior, Mucoadhesion, and In Vitro Drug Release
Source: Gels. 2026 Jan 19;12(1):90. doi: 10.3390/gels12010090 (PMC12841464; doi:10.3390/gels12010090)
Supplement: Supplementary file 1 [file gels-12-00090-s001.zip › gels-3991505-supplementary.pdf]

Supplementary Materials

pH-Sensitive Dextrin-Based Nanosponges Crosslinked with Pyromellitic Dianhydride and Citric Acid: Swelling, Rheological Behavior, Mucoadhesion, and In Vitro Drug Release

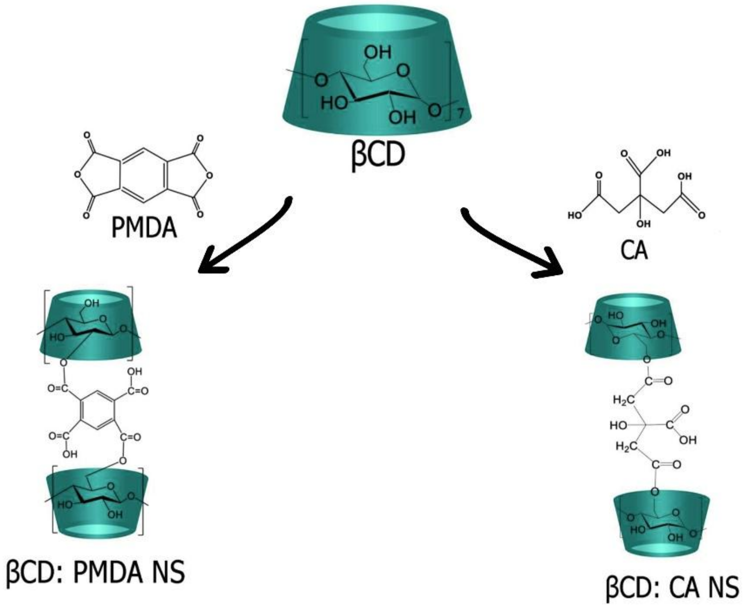

Figure S1. Schematic representation of the synthesis of β-CD: PMDA and β-CD: CA nanosponges (NS).

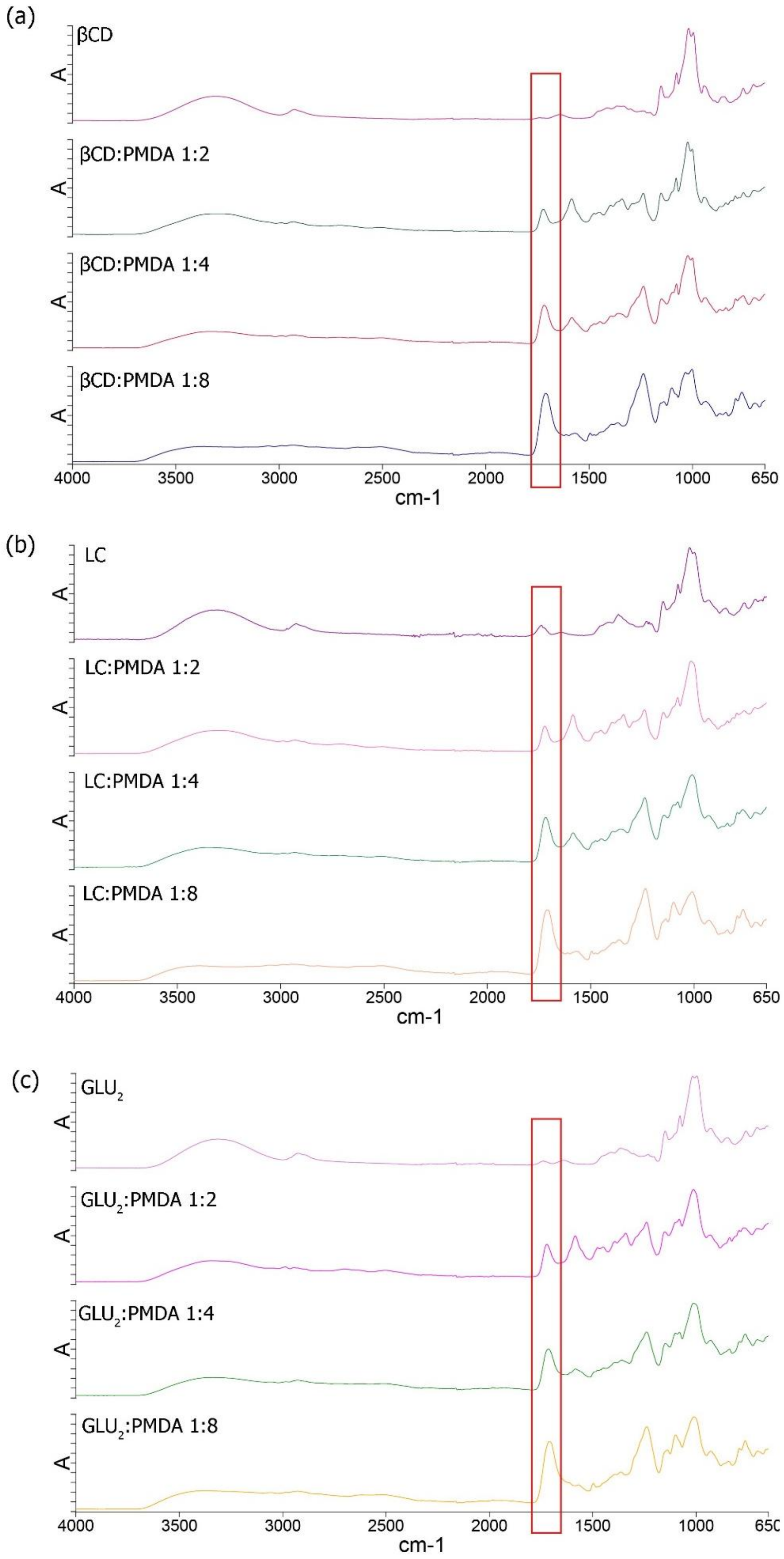

Figure S2. FTIR spectra of PMDA-based D-NS: (a) β-CD:PMDA NS, (b) LC:PMDA NS, c) Glu_2: PMDA NS.

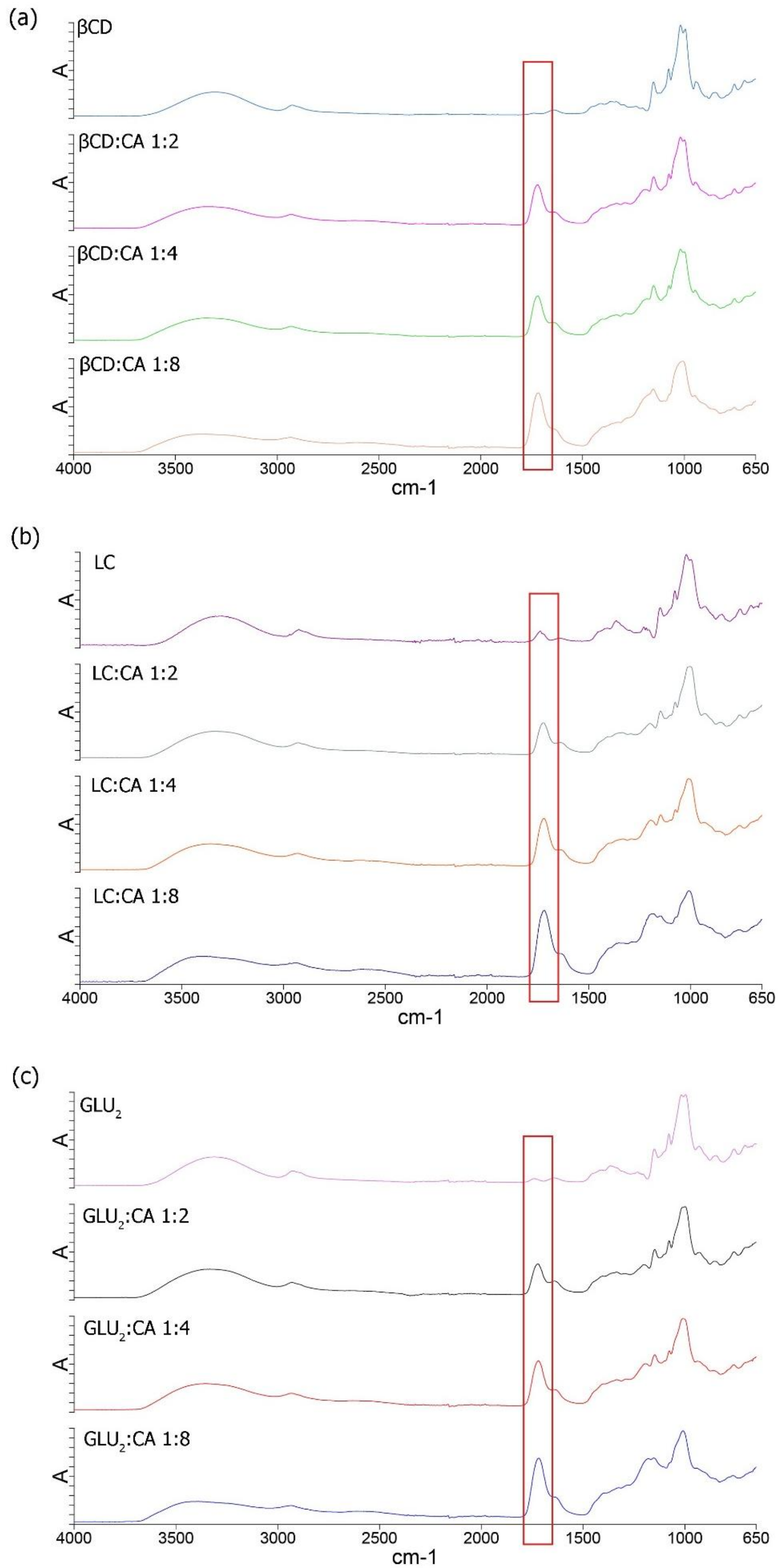

Figure S3. FTIR spectra of CA-based D-NS: (a) β-CD:CA NS, b) LC:CA NS, c) Glu_2:CA NS.

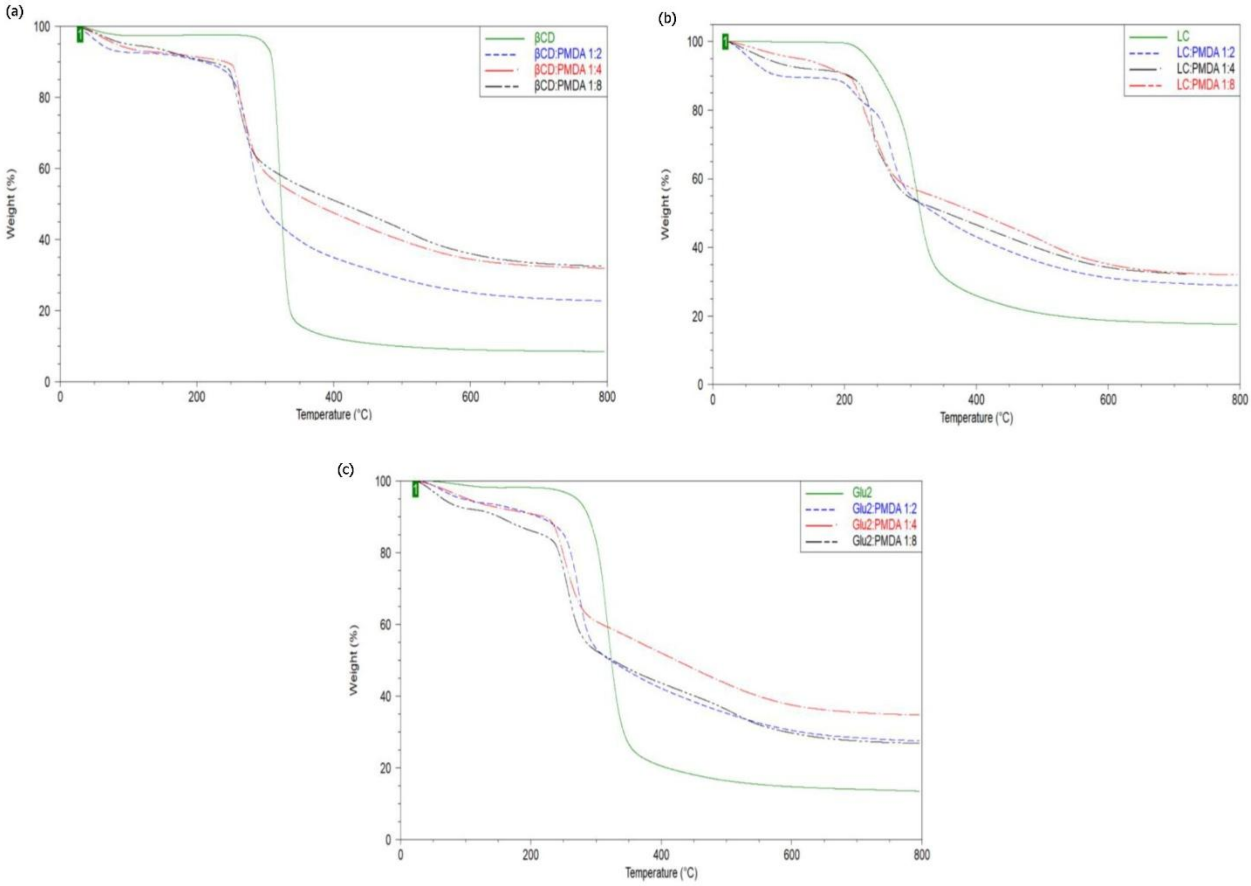

Figure S4. TGA curves of PMDA-based D-NS: (a) β-CD:PMDA NS, (b) LC:PMDA NS, c) Glu_2: PMDA NS.

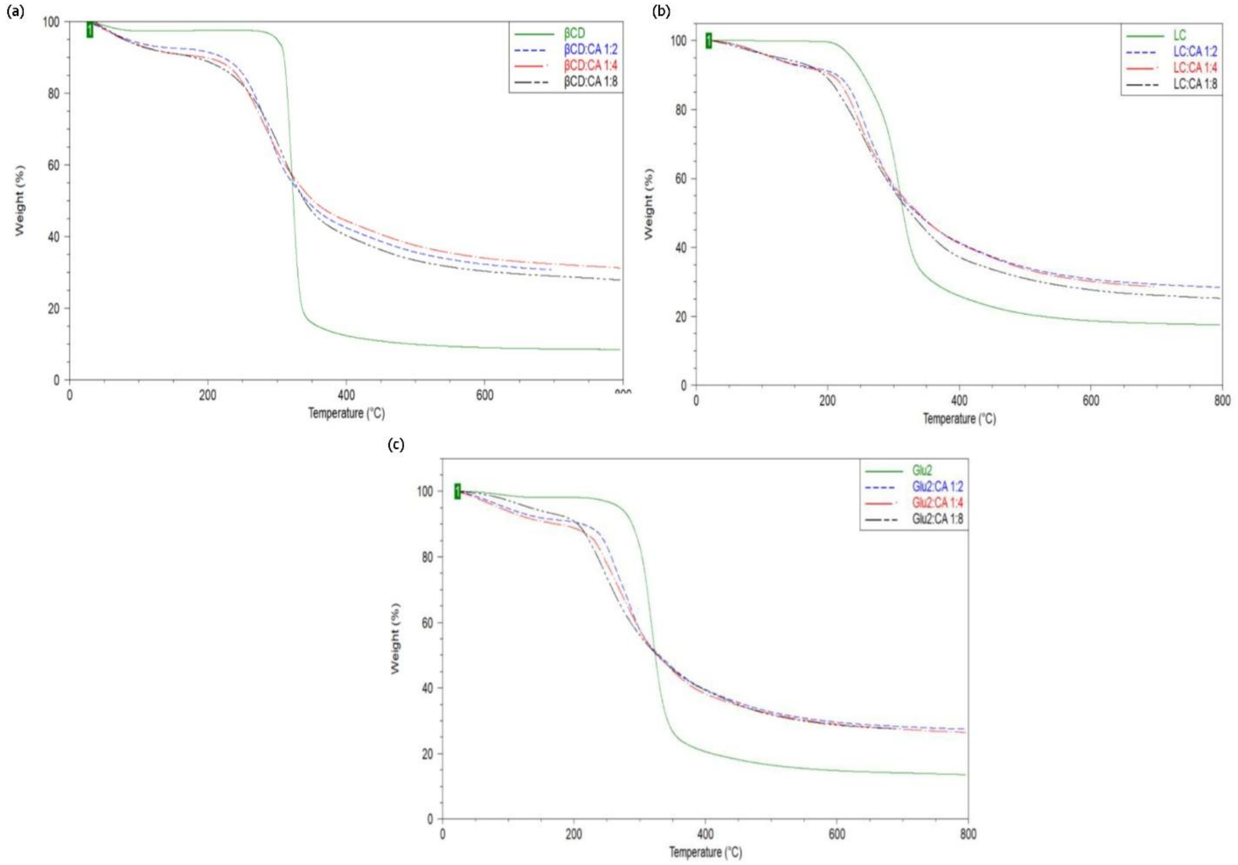

Figure S5. TGA curves of CA-based D-NS: (a) β-CD:CA NS, b) LC:CA NS, c) Glu_2:CA NS.

(a)

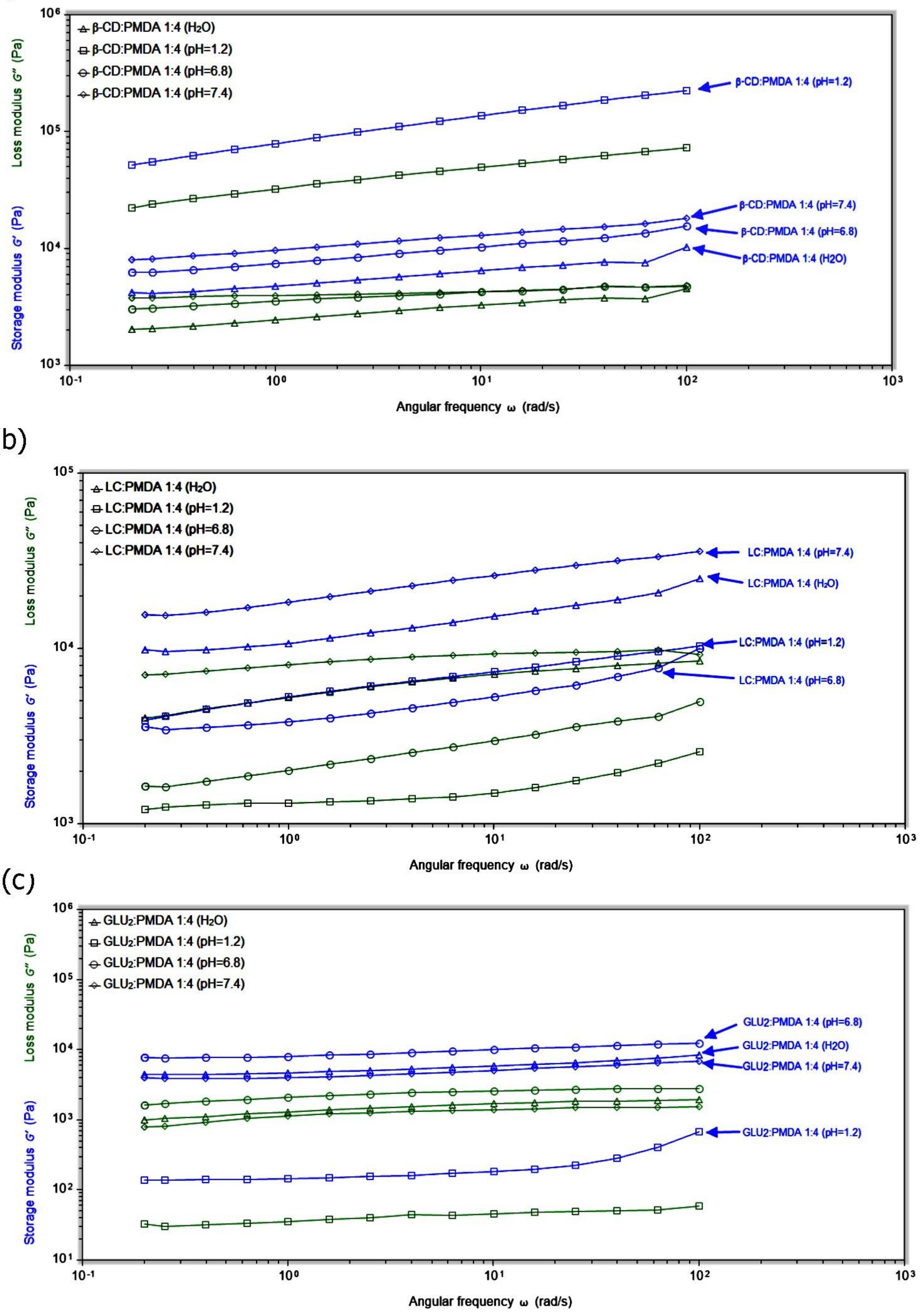

Figure S6. Storage (G′) and loss (G′′) modulus versus angular frequency for (a) β-CD:PMDA 1:4 NS; (b) LC:PMDA 1:4 NS; and (c) Glu_2:PMDA 1:4 NS; 0.8 mm gap size. Nanocarriers were allowed to swell in deionized water and in aqueous solutions at pH 1.2, 6.8, and 7.4.

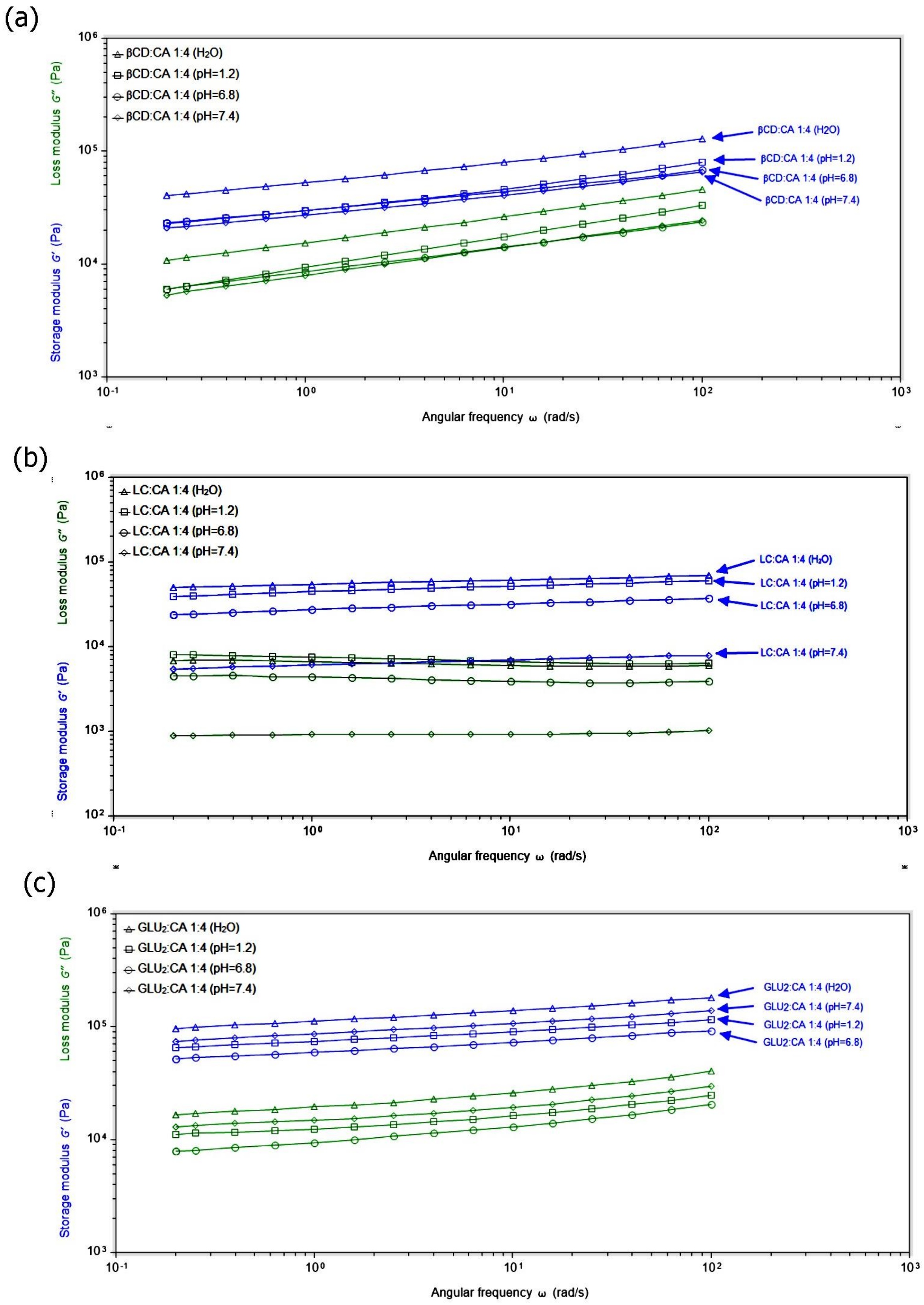

Figure S7. Storage (G′) and loss (G′′) modulus versus angular frequency for (a) β-CD:CA 1:4 NS; (b) LC:CA 1:4 NS; and (c) Glu_2:CA 1:4 NS; 0.8 mm gap size. Nanocarriers were allowed to swell in deionized water and in aqueous solutions at pH 1.2, 6.8, and 7.4.

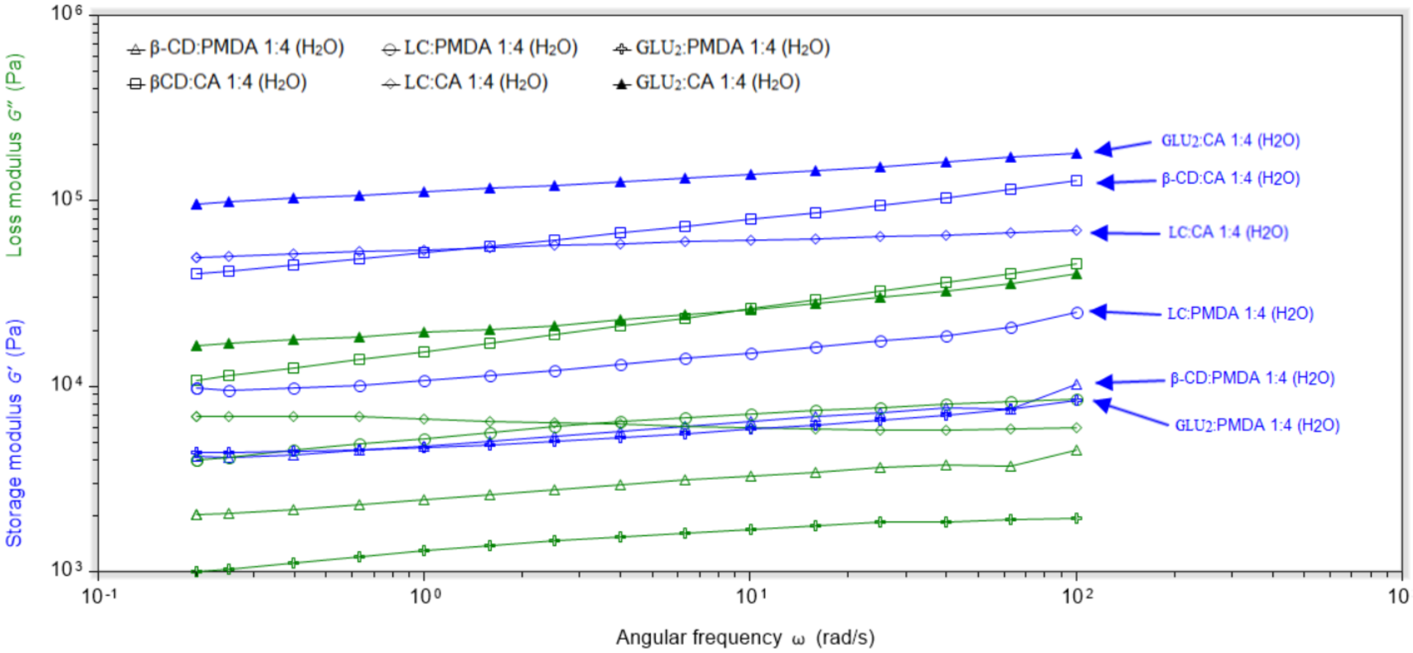

Figure S8. Storage (G′) and loss (G′′) modulus versus angular frequency for β-CD: PMDA 1:4 NS; β-CD: CA 1:4 NS; LC: PMDA 1:4 NS; LC: CA 1:4 NS; Glu2:PMDA 1:4 NS; Glu2:CA 1:4 NS; 0.8 mm gap size. Nanocarriers are swollen in deionized water.

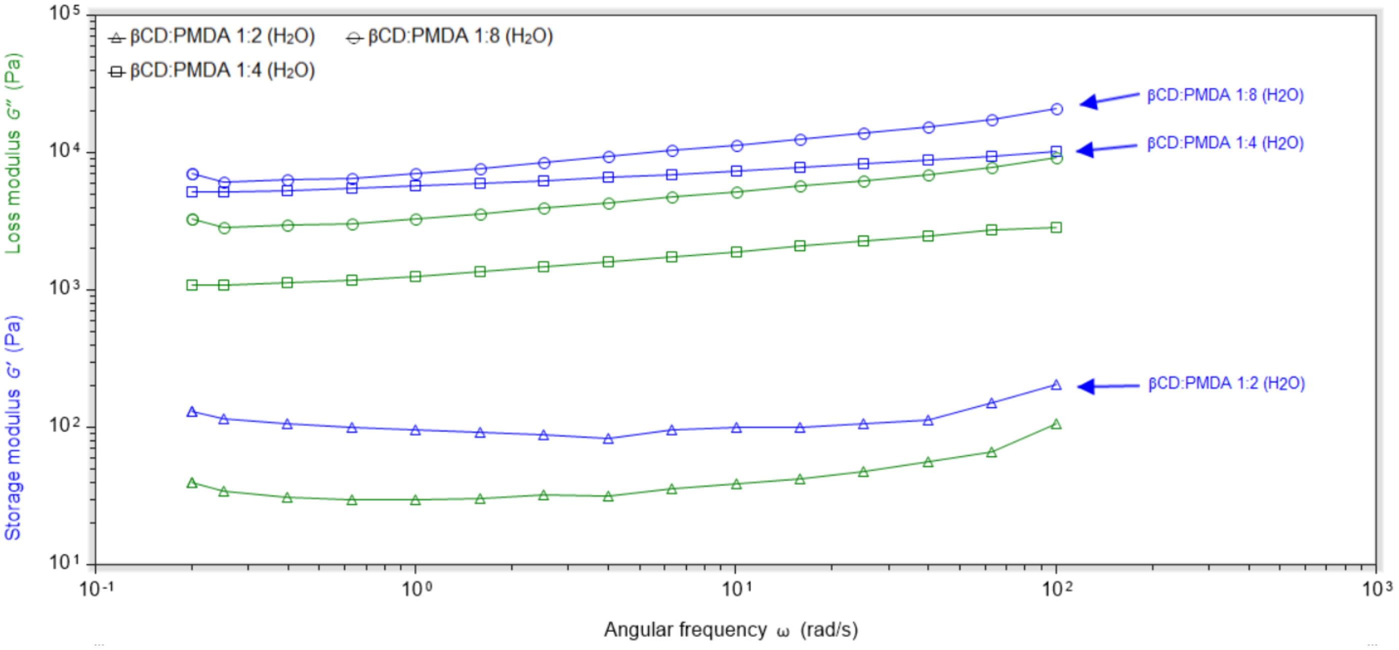

Figure S9. Storage (G′) and loss (G′′) modulus versus angular frequency for β-CD: PMDA 1:2 NS; β-CD: PMDA 1:4 NS; and β-CD: PMDA 1:8 NS; 0.8 mm gap size. Nanocarriers are swollen in deionized water.
